# Understanding activity-stability tradeoffs in biocatalysts by enzyme proximity sequencing

**DOI:** 10.1038/s41467-024-45630-3

**Published:** 2024-02-28

**Authors:** Rosario Vanella, Christoph Küng, Alexandre A. Schoepfer, Vanni Doffini, Jin Ren, Michael A. Nash

**Affiliations:** 1 Institute of Physical Chemistry, Department of Chemistry, University of Basel, 4058 Basel, Switzerland; 2 Department of Biosystems Science and Engineering, ETH Zurich, 4058 Basel, Switzerland; 3 Institute of Chemical Sciences and Engineering, École Polytechnique Fédérale de Lausanne (EPFL), 1015 Lausanne, Switzerland; 4 National Center for Competence in Research (NCCR), Catalysis, École Polytechnique Fédérale de Lausanne (EPFL), 1015 Lausanne, Switzerland; 5 National Center for Competence in Research (NCCR), Molecular Systems Engineering, 4058 Basel, Switzerland; 6 Swiss Nanoscience Institute, 4056 Basel, Switzerland

**Keywords:** Oxidoreductases, Biophysical chemistry, Biocatalysis, Chemical engineering

## Abstract

Understanding the complex relationships between enzyme sequence, folding stability and catalytic activity is crucial for applications in industry and biomedicine. However, current enzyme assay technologies are limited by an inability to simultaneously resolve both stability and activity phenotypes and to couple these to gene sequences at large scale. Here we present the development of enzyme proximity sequencing, a deep mutational scanning method that leverages peroxidase-mediated radical labeling with single cell fidelity to dissect the effects of thousands of mutations on stability and catalytic activity of oxidoreductase enzymes in a single experiment. We use enzyme proximity sequencing to analyze how 6399 missense mutations influence folding stability and catalytic activity in a D-amino acid oxidase from *Rhodotorula gracilis*. The resulting datasets demonstrate activity-based constraints that limit folding stability during natural evolution, and identify hotspots distant from the active site as candidates for mutations that improve catalytic activity without sacrificing stability. Enzyme proximity sequencing can be extended to other enzyme classes and provides valuable insights into biophysical principles governing enzyme structure and function.

## Abstract (web summary)

Understanding the complex relationships between enzyme sequence, folding stability and catalytic activity is essential for applications, but current technologies cannot simultaneously resolve both stability and activity phenotypes and couple these to gene sequences at large scale. Here, the authors report Enzyme Proximity Sequencing (EP-Seq), a deep mutational scanning method to assay both expression level and catalytic activity of thousands of oxidoreductase variants from a cellular pool in a single experiment.

## Introduction

Soluble proteins produced through natural selection are typically only marginally stable. For enzymes, local flexibility is required at the active site to achieve catalytic activity, however excessive mobility renders them susceptible to denaturation. This tradeoff between activity and stability is still not well understood in protein science^1–4^.

Observations on small numbers of homologous sequences have shown how cold-adapted enzymes are typically more active than thermophilic homologs^5–7^, however, competing studies relying on database meta-analysis^8^, experimental data and comparative phylogenetics^9^ have challenged this thermal rate compensation model. The question of how enzymes encode activity and stability, and the interplay between the two over the course of natural evolution or during experimental laboratory directed evolution^10,11^ therefore remains open. For enzyme engineering, these tradeoffs mean that both stability and activity cannot be optimized simultaneously or with equal success. If we could experimentally determine sequence-function relationships describing folding stability and catalytic activity of enzyme variants at large scale, it would enable an understanding of the activity-stability tradeoff, and potentially unlock enhanced enzymes for industrial and biomedical applications.

With the advent of deep mutational scanning (DMS), the effects of large numbers of genetic mutations on protein phenotype can be analyzed using massively parallel methods^12–14^.

However, catalytic enzymes are challenging for DMS studies because very few massively scalable screening methods which successfully link genotype and phenotype exist. Typically, enzyme fitness is coupled to host cell survival using growth-based selection. Alternatively, microdroplet methods allow single clones to be analyzed using colorimetric assays followed by droplet sorting and sequencing. Enrichment of variants from pre- to post-selected pools allows tabulation of phenotypic scores and provides insights into mutational fitness landscapes^15–21^. In nearly all prior implementations of DMS on enzymes, however, fitness scores comprise a conflation of expression level (i.e. enzyme abundance) and catalytic activity. Klesmith and colleagues showed how enzyme fitness scores determined through growth-based selection could be combined with solubility scores from independent assays to reveal evolutionary origins of stability activity trade-offs^22^. Markin et al. further presented a microfluidic enzyme expression platform which decoupled the catalytic properties of each variant from their expression levels^23^. While very powerful, each platform has associated limitations in terms of throughput, ease-of-use, compatible chemistries, and robustness.

Here we report enzyme proximity sequencing (EP-Seq), a novel DMS-based method that combines enzyme proximity labeling with next-generation DNA sequencing (NGS) technology to assay both expression level and catalytic activity phenotypes of thousands of variant enzymes from a cellular pool in a single experiment. EP-Seq leverages features of yeast surface display^24,25^ to measure the expression levels of each enzyme variant via a pooled cell sorting-sequencing experiment. In parallel, phenoxyl radical-based^26–29^ cell surface proximity labeling links enzyme activity to a fluorescent signal, which is then quantified by sorting and sequencing. We used EP-Seq to study a nearly comprehensive site saturation mutational library of D-amino acid oxidase (DAOx) from the yeast *Rhodotorula gracilis*, a model flavoprotein with industrial and therapeutic applications^30–36^. Downstream computational analysis of EP-Seq data reveals rich biophysical insights into the enzyme by quantifying fitness propensities of substituted residues, identifying protein regions where catalytic activity served as an evolutionary constraint on folding stability, and making predictions of catalysis-enhancing mutations that maintain folding stability.

## Results

### Workflow overview

**Fig. 1:**
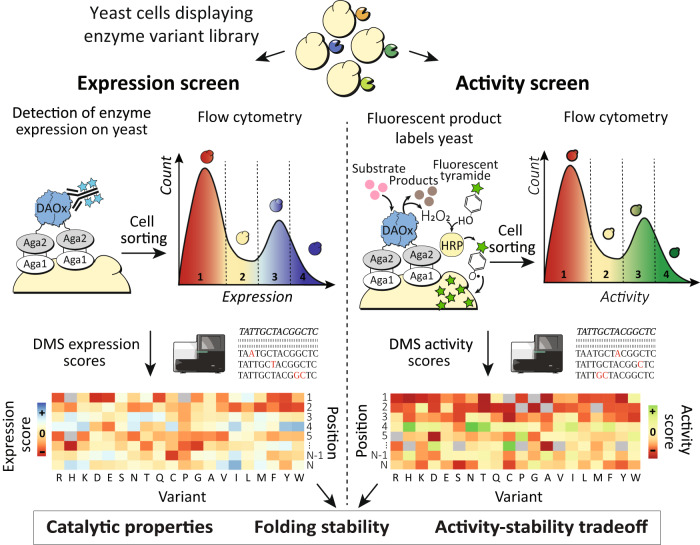
Schematic depicting the EP-Seq workflow. (Top) A pooled library of enzyme variants is displayed on yeast. (Left) The cell population is sorted into bins based on the expression level of the displayed enzyme. (Right) The pooled variant library is assayed for DAOx activity using a cascade peroxidase-mediated proximity labeling reaction with single cell fidelity and sorted into bins. The genetic composition of cells in the sorted bins is quantified via high-throughput sequencing and the distribution of each variant along the expression and activity axes is converted into a fitness score. Joint analysis of the two independent datasets provides insights into the effects of mutations on folding stability and activity of the enzyme.

An overview of the EP-Seq workflow is shown in Fig. 1. In one branch of the experiment (Fig. 1, left), we analyze the expression levels of thousands of variants in parallel by displaying a variant library on yeast, staining the expressed proteins with fluorescent antibodies, and sorting the cells into 4 bins using fluorescence activated cell sorting (FACS). We then use NGS to sequence the variants in each bin, and convert the NGS reads into expression fitness scores using a custom computational pipeline. There is strong evidence that the level at which yeasts secrete and display a given protein sequence is correlated with its folding stability^22,25^. Destabilizing mutations can activate the yeast quality control system, exposing variants to proteases in the secretory pathway which degrade unstable sequences and lower expression levels^37^. The expression level of a variant enzyme analyzed through FACS and DMS can therefore serve as a proxy for folding stability. In this study, we use the term folding stability to describe the impact of mutations on the overall cellular stability of the target protein. This primarily relates to structural and thermodynamic stability, but can also include other factors like mRNA stability, efficiency of translation and secretion, and susceptibility to protease degradation, all of which contribute to changes in the protein’s expression level.

In a parallel branch of the experiment (Fig. 1, right), the oxidase activity of enzyme variants is assayed at large scale in a pooled format using a horseradish peroxidase (HRP)-mediated phenoxyl radical coupling reaction at the yeast surface. Similar proximity labeling reaction schemes relying on HRP or ascorbate peroxidase-2 (APEX2) have been used in quantitative proteomics^38^, proximity labeling in live cells^39–42^, electron microscopy labelling^43,44^ and signal amplification in biosensors^45,46^. In EP-Seq, the short half-life of phenoxyl radicals limits the labeling reaction to the surface of the cell that generates H_2_O_2_, affording an artificial quasi single-cell reaction compartment by means of a reaction-diffusion limitation. The pooled cells displaying the variant library stained in this fashion are sorted into bins based on fluorescent intensity, and sequenced by NGS. Finally, the large datasets obtained in the two parallel screening protocols are combined, cross-referenced and studied to reveal biophysical insights into protein sequence, stability, and function.

### Quantifying DAOx stability and catalytic activity fitness with EP-Seq scores

We applied the workflow described above to study the 80 kDa homodimeric flavin adenine dinucleotide (FAD) dependent DAOx from *Rhodotorula gracilis* (Rg), which promiscuously catalyzes deamination of D-amino acids to alpha keto acids, generating H_2_O_2_ as a byproduct^35^. DAOx has attracted attention for applications in both industry and biomedicine, for example, in resolving racemic amino acid mixtures, producing antibiotics^32^ or as a proposed cancer therapy via reactive oxygen species^30,31,47^. We first optimized the functional expression and display of the wild type DAOx (WT DAOx) fused with the Aga2 anchor protein and established the tyramide-based proximity labeling assay to detect the activity of each displayed enzyme with single-cell fidelity^26,48,49^ (Supplementary Note 1). Next, we constructed a library for DMS analysis through site saturation mutagenesis over the entire coding region of DAOx and assigned 15 nucleotides unique molecular identifier (UMIs) to each DAOx variant of the library (Supplementary Note 2).

**Fig. 2:**
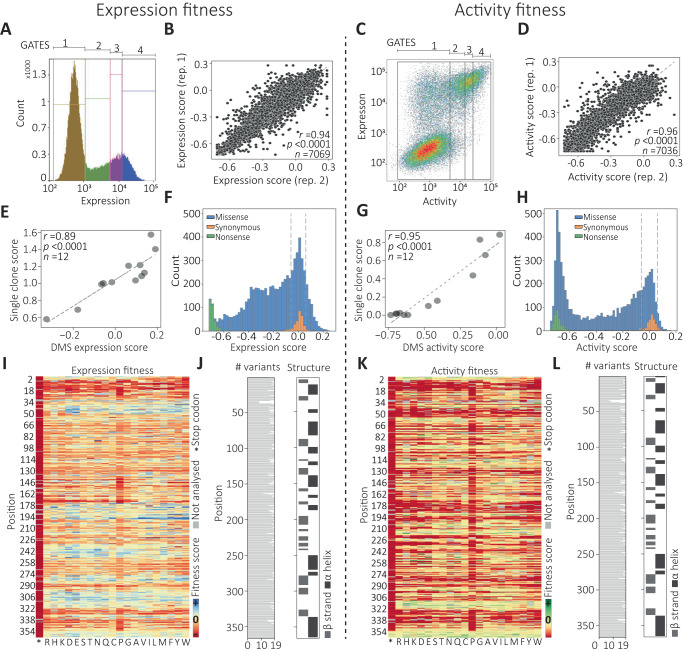
Deep mutational scanning of DAOx expression and catalytic activity by EP-Seq. **A** Sorting gates for analysis of display levels. **B** Linear regression (Pearson *r*, two-tailed) between expression scores calculated from two biological replicates. **C** Sorting gates for catalytic activity screening. **D** Linear regression (Pearson *r*, two-tailed) between activity scores calculated from two biological replicates. **E** Linear regression (Pearson *r*, two-tailed) between variant surface display level measured in monogenic yeast culture vs. DMS expression fitness analyzed by EP-Seq for 12 DAOx single mutant variants. **F** Distribution of expression fitness effects measured by EP-Seq. Dashed lines represent the range of fitness score for synonymous variants. **G** Linear regression (Pearson *r*, two-tailed) between variant activity level measured in monogenic yeast culture via peroxidase assay (Amplex Red) vs. DMS activity fitness analyzed by EP-Seq for 12 DAOx single mutant variants. **H** Distribution of activity fitness effects measured by EP-Seq. Dashed lines represent the range of fitness score for synonymous variants. **I** Expression fitness scores for each variant represented as a heatmap. **J** Number of variants analyzed per position in the expression dataset, and secondary structure classification per position (PDB: 1C0P). **K** Activity fitness scores for each variant obtained by EP-Seq represented as a heatmap. **L** Number of variants analyzed per position in the activity dataset and secondary structure classification per position (PDB: 1C0P). Links to interactive and color blind accessible heatmaps can be found in the data availability statement section of the manuscript.

We investigated the effects of single amino acid substitutions on DAOx expression and display at the yeast surface (Fig. 2, left). Following induction (48 h, 20 °C, pH 7), we stained the C-terminal His-tag of the DAOx variant library with primary and fluorescent secondary antibodies. We sorted the library into 4 bins based on expression level, where the non-expressing bin was set using a negative control cell population incubated with only the secondary antibody. The remaining yeasts were sorted into three sub-populations containing equal percentages of expressing cells (Fig. 2A). After sorting, we extracted plasmid DNA from each sorted cell population, PCR amplified the regions corresponding to the UMIs, and sent the amplicons for single end (SE100) Illumina sequencing on a NovaSeq 6000. The number of reads per sample was on average 25-fold higher than the number of cells sorted into the corresponding FACS bin (Supplementary Table 2). We filtered the UMI sequences by read quality (Phred score ≥ Q20) and expected size (15 nucleotides) before assigning them to the corresponding DAOx variants using the look-up table. We converted the number of reads per variant into number of cells (Methods, Eq. 1) and calculated a final expression score (Exp) for each variant (Methods, Eq. 2). The fitness score per variant was then calculated as ${\log }_{2}({\beta }_{{{{{{\rm{v}}}}}}}/{\beta }_{{wt}})$ where ${\beta }_{{{{{{\rm{v}}}}}}}$ was the weighted mean expression score of the variant enzyme and ${\beta }_{{wt}}$ was the score of WT DAOx (Methods, Eq. 3).

To analyze DAOx catalytic activity in a massively parallel fashion, we used a reaction cascade that converts DAOx enzymatic activity into a fluorescent label on the cell wall. This approach is related to prior work from our lab and others on enzyme-mediated polymerization and peroxidase-based proximity labeling^14,27–29,50,51^. We set the low fluorescence gate to include entirely the population of not displaying cells or displaying inactive variants (Fig. 2C, Gate 1). The remaining cells were equally divided into three populations corresponding to increasing levels of tyramide-488 signal (Fig. 2C). We determined the genetic sequences and their relative abundance in each bin through Illumina sequencing as described above, and identified the corresponding mutant enzyme sequences using the look-up table. The activity score (Act) per variant was calculated (Methods, Eq. 2) and the activity fitness score was computed by using as reference the score of the wild type DAOx (Methods, Eq. 3). In both the expression and activity screens, we determined a consensus score for each mutant (Methods, Eq. 4) and we assessed the reproducibility of the DMS workflow by calculating Pearson’s *r* value for a linear regression of the fitness scores measured in two biological replicates and represented by at least 10 total cells. For the expression assay replicates, the *r* value was 0.94 (*n* = 7069; *p* < 0.0001), and for the activity assay replicates, it was 0.96 (*n* = 7036; *p* < 0.0001) (Fig. 2B, D).

### Validation of EP-Seq scores

To validate the DMS fitness scores of single clones, we randomly selected 12 variants and tested them individually using bulk expression and activity assays. The expression levels of the variants measured individually using yeast display and flow cytometry strongly correlated with expression fitness scores obtained from DMS (*r* = 0.89, *p* < 0.0001) (Fig. 2E). The activity levels of the same 12 single mutant variants were next measured using an Amplex Red assay, and the initial reaction rate was measured for the mutants and the wild type enzymes. The single clone score for each mutant was obtained by dividing initial reaction rate of the mutant by that of wild type. All mutants tested showed activity levels consistent with those obtained from EP-Seq (*r* = 0.95, *p* < 0.0001) (Fig. 2G).

To further validate the observed scores, we plotted a histogram to visualize the expression (Fig. 2F) and activity (Fig. 2H) fitness scores of all single nucleotide variants in the DAOx gene and color coded them to distinguish between missense, nonsense (i.e. stop codon), and synonymous mutations. We found that the distribution of missense variants scores for both expression (*n* = 6434 variants) and activity (*n* = 6404 variants) fitness were broadly distributed (Exp min = −0.72, Exp max = 0.25, Act min = −0.75, Act max = 0.16). Fitness score distributions were on average offset towards negative fitness values, indicating a generally deleterious effect of mutation on both expression and activity (median Exp = −0.15, median Act = −0.29, Fig. 2F, H). The distribution of fitness scores containing synonymous mutations in the expression screen was centered at 0.00 ± 0.057 (*n* = 301 variants), while in the activity screen it was centered at 0.00 ± 0. 056 (*n* = 300 variants). This range of fitness values defined a neutral fitness range of the assay. Variants whose scores fell in this range were expected to have fitness comparable to that of wild type. Additionally, mutants with nonsense mutations (i.e. stop codons) had strong negative fitness on both expression and activity, with average expression score of −0.69 ± 0.04 (*n* = 334) (Fig. 2F) and average activity score of −0.67 ± 0.12 (*n* = 332). The higher activity scores and standard deviation of nonsense variants compared to expression scores of the same variants are due to the effect of stop codon at C-terminal positions of the enzyme (position>350) preserving the formation of a fully folded and functional DAOx enzyme.

As further validation, we calculated the average expression and activity scores per position (*n* = 364) and mapped the values onto the DAOx structure (PDB: 1C0P) (Supplementary Fig. 4A, B). We tested for EP-Seq score correlation with a protein stability prediction algorithm (FOLDX)^52^ (Methods, Eq. 5). We used FOLDX to calculate the mean ΔΔG score per position of the protein (*n* = 354) and compared them to expression fitness scores obtained from the left branch of EP-Seq. We found strong correlations between EP-Seq expression scores and predicted ΔΔG values (Exp rho = −0.51 *p* < 0.0001, Supplementary Fig. 4C), supporting the use of yeast surface display for evaluating the effects of mutations on thermodynamic folding stability. We further compared the predicted ΔΔG values to EP-Seq catalytic activity scores per position of DAOx and again found significant correlation

(Act rho = −0.59 *p* < 0.0001, Supplementary Fig. 4D). As a baseline level of structural stability is required for an enzyme to be catalytically active, this result supports that part of the EP-Seq activity score is attributable to effects of mutations on the folding stability (and therefore expression) of DAOx.

### Mutability landscapes of DAOx stability and catalytic activity

We visualized the mutational effects of the 6768 (6434 missense, 334 nonsense) and 6736 (6404 missense, 332 nonsense) mutations in the expression and activity screens (respectively) as fitness heatmaps (Fig. 2I, K). The numbers of missense variants corresponded to 93% and 92.5% (respectively) of all possible single amino acid substitutions in DAOx (Fig. 2J, L). The expression heatmap (Fig. 2I) reveals patterns of higher and lower tolerance for mutation along the DAOx sequence, discussed in detail below. The N-terminal region (residues 8–32) was found to be highly intolerant to amino acid substitutions. This suggests it plays a role in folding stability and could act as an N-terminal intra-molecular chaperone^53^. The Rossmann fold is highly conserved in this region, making contact with the FAD cofactor, stabilizing structure and maintaining function of the enzyme^35^.

### Biophysical properties shape both expression and activity landscapes

**Fig. 3:**
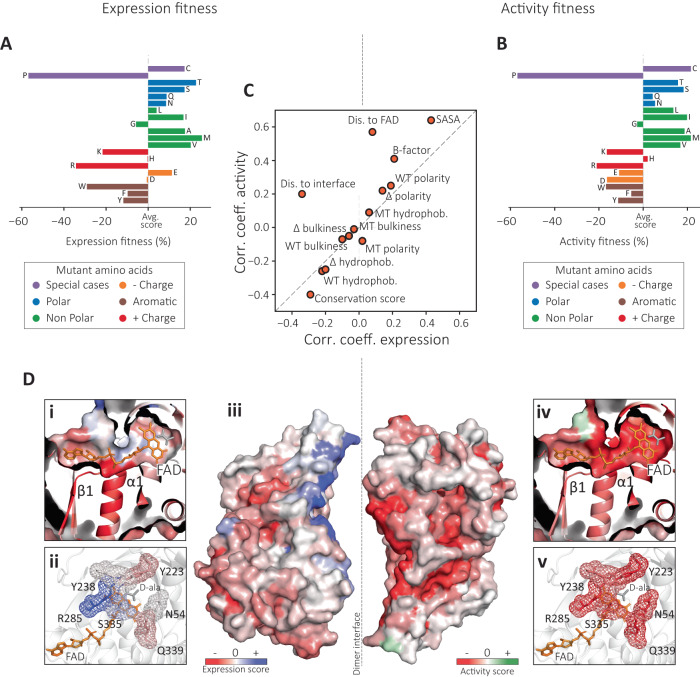
EP-Seq reveals biophysical properties that differentially influence expression and activity. **A** Relative impact of mutant residue identity on DAOx expression. **B** Relative impact of mutant residue identity on the DAOx enzymatic activity. **C** Plot of Pearson correlation coefficients (*r*) calculated for expression (*x*-axis) or activity (*y*-axis) fitness scores with respect to several biophysical properties of mutated sites in DAOx. **D** DAOx structure colored by expression or activity fitness scores: (i) Residues surrounding FAD cofactor colored by expression score; (ii) Residues contacting the substrate D-alanine colored by expression score; (iii) Expression (left) or activity (right) scores used to color the surface of DAOx monomers. The monomers are shown separated from each other to improve visibility of the interface; (iv) Residues surrounding FAD cofactor colored by activity score; (v) Residues contacting the substrate D-alanine colored by activity score.

We evaluated the effect of mutant residue identity on both expression and activity of DAOx using data from 6399 single missense variants for which both EP-Seq expression and activity scores were available (Fig. 3A, B, Supplementary Table 4, Supplementary Note 3). We observed that the introduction of proline had the most pronounced negative impact on both expression and activity, particularly when located in the α helix and β sheet regions of DAOx^54,55^ (Supplementary Fig. 5A, B, Supplementary Note 4). The introduction of cysteine, neutral polar and non-polar residues (excluding glycine) had a positive impact on the studied properties, while the introduction of charged residues (excluding histidine) resulted in a lower activity score than average. Residues with negative charges, glutamic and aspartic acids, exhibited an overall neutral to positive effect on the expression score compared to the average effect of all substituted residues. Finally, among hydrophobic residues, introduction of bulky tryptophan impaired both expression and activity fitness (Fig. 3A, B, Supplementary Table 4). As a general trend we also observed that DAOx linker regions had greater tolerance to mutations in both expression and activity screens compared to structured regions of the same enzyme (Supplementary Fig. 5C, D, Supplementary Note 4). We next analyzed expression and activity fitness as a function of the substituted wild type amino acids (Supplementary Fig. 5E, F, Supplementary Note 5) and found the substitution of hydrophobic core aromatic residues together with valine, which is one of the most abundant residues in DAOx (*n* = 27), to have the highest negative impact on the expression and activity of the enzyme. On the contrary, mutation of polar and charged amino acids was associated with on average higher scores than the other classes of residues (Supplementary Fig. 5E, F, Supplementary Note 5).

Next, we assigned residue specific scores for hydrophobicity, bulkiness and polarity of wild type and mutant amino acids^56^ to each of the 6399 single missense mutations registered by both assays and calculated a Pearson linear correlation coefficient *r* between the values of each feature and the experimental fitness scores (Fig. 3C, Supplementary Fig. 6A). We observed no or very low linear correlation between the experimental scores and properties of mutant residues (Mut. hydrophobicity, Mut. polarity, Mut. bulkiness, Supplementary Fig. 6A). Consistent with our previous observations, the hydrophobicity of wild type residues negatively correlated with both expression (*r* = −0.22, *p* < 0.0001) and activity scores (*r* = −0.26, *p* < 0.0001), suggesting low tolerance for mutations in the protein core (WT, Δ hydrophobicity, Fig. 3C, Supplementary Fig. 6A). We found moderate negative correlation of both expression and activity datasets when considering the size of wild type amino acids or the size difference between wild type and mutant residue side chains (Fig. 3C, Supplementary Fig. 6A, WT and Δ bulkiness). Finally, a positive correlation of expression (*r* = 0.19, *p* < 0.0001) and activity scores (*r* = 0.25, *p* < 0.0001) with the polarity of wild type amino acids indicated higher tolerance for mutation of polar residues, typically found on the surface of the protein (Fig. 3C, Supplementary Fig. 6A, WT and Δ polarity).

### Identification of stability activity trade-off regions in DAOx

We next sought to determine whether our expression and activity datasets could be used to identify regions of stability-activity tradeoffs. We analyzed average expression and activity scores of DAOx at each position (*n*_Epx_ = *n*_Act_ = 360) in relation to biophysical properties of the mutated sites (Fig. 3C) and observed several trends. For example, both expression and activity fitness scores were positively correlated with solvent accessible surface area (SASA) of the mutated site (Exp *r* = 0.43, *p* < 0.0001; Act *r* = 0.64, *p* < 0.0001). The temperature factor (B-factor) was similarly positively correlated with both fitness scores (Exp *r* = 0.21, *p* < 0.0001; Act *r* = 0.41, *p* < 0.0001) indicating higher mutational tolerance at sites located at the protein surface and at sites with high structural mobility (Fig. 3C, Supplementary Fig. 6B, C).

We analyzed fitness scores of mutated sites with respect to their distance from the FAD cofactor (Fig. 3C, Dis. to FAD). We found that mutations near FAD often negatively impacted the catalytic activity of the enzyme, as indicated by the positive correlation between the activity scores and the distance of the mutated residue from FAD (*r* = 0.57, *p* < 0.0001, Supplementary Fig. 6D bottom). In contrast, expression fitness scores were not significantly correlated with distance of the mutated residue to FAD (*r* = 0.08, *p* = 0.1134, Supplementary Fig. 6D top), suggesting that folding stability was insensitive to this parameter. We color-coded the 3D structure of DAOx according to activity and expression scores in the vicinity of the FAD cofactor and in the active site (Fig. 3D i, ii, iv, v), where we found the largest differences between the activity and expression scores. Mutations in close proximity to FAD (distance<4 Å) greatly impaired the catalytic activity of the enzyme (avg. Act = −0. 557, −66%, *n* = 387; avg. Act all = −0.336, *n* = 6,399; Fig. 3D iv) while at the same positions the expression scores showed the opposite trend. This revealed how mutations at the catalytic site tend to harm activity but improve stability (avg. Exp = −0.149, *n* = 387, +17%, avg. Exp all = −0.180, *n* = 6,399; Fig. 3D i), supporting a well-documented phenomenon on the thermodynamic price paid by an enzyme to remain catalytically active under conditions of functional selection^4^.

A similar behavior was found for residues known to coordinate the substrate D-alanine^35^. While mutating these residues impaired enzyme activity (avg. Act = −0.457, −36%, *n* = 111; avg. Act all = −0.336, *n* = 6399; Fig. 3D v), it tended to improve stability (avg. Exp = −0.06, +60%, *n* = 111; avg. Exp all = −0.180, *n* = 6399; Fig. 3D ii). Among the residues interacting with FAD, the loop between strand β1 and helix α1 is highly conserved among Rossmann folds (GSGVIGL, positions: 11-17) and is characterized by negative fitness scores in both datasets (avg. Exp = −0.479, avg. Act = −0.639) (Fig. 3D i, iv). This indicates both a functional and structural role of FAD in the overall fitness of the enzyme. These results suggest DAOx folding is facilitated by interactions with FAD, which is also essential for its catalytic activity. The strong binding affinity between DAOx and FAD (dissociation constant, *K*_D_ = 20 nM) and the low abundance of apo-enzyme further support these observations^57,58^.

### Dimerization stabilizes marginally stable but functional DAOx monomers

DAOx is active as a homodimer in its native state and dimerization is thought to be required for activity^58,59^. This motivated a detailed analysis of mutations in close proximity to the dimer interface, and whether they could significantly change folding stability and catalytic activity of the enzyme. We calculated the distance between each residue and the closest residue found at the dimer interface, and assigned this distance value to each single missense mutation found in both the expression and activity screens. We then calculated a linear correlation coefficient between the experimental fitness scores and the distance values. We found that the activity fitness data showed a positive correlation with the distance of the mutated site from the dimer interface (*r* = 0.20, *p* < 0.0001, Fig. 3C, Dis. to interface, Supplementary Fig. 6E bottom), while the expression scores correlated negatively with it (*r* = −0.34, *p* = <0.0001, Fig. 3C, Dis. to interface, Supplementary Fig. 6E top). On average, sites located closer to the dimer interface were more tolerant to mutation in the expression screen, but less in the activity screen. This reflects a scenario where the enzyme tolerates some amount of instability in order to remain as an active dimer, analogous to the effects at the catalytic site. We visualized these effects on the surface of the 3D structure of the DAOx dimer (Fig. 3D iii). Mutations located near the dimer interface (distance < 5 Å) impaired the catalytic activity of the DAOx enzyme as indicated by the dominant red color of the structure (avg. Act = −0.363, −7%, *n* = 1298, avg. Act all = −0.336, *n* = 6,399; Fig. 3D iii, right). The same mutations showed overall positive effects on the expression and stability of the DAOx structure (avg. Exp = −0. 07, +61%, *n* = 1298; avg. Exp all = −0.180, *n* = 6399; Fig. 3D iii, left). We attributed this behavior to a strong dependency of DAOx catalytic activity on dimerization^58,59^. Apparently, dimerization along with the conformation of the catalytic site provide functional constraints during evolution of this enzyme, suggesting the evolution of a more stable globular and monomeric form of DAOx might have been impeded during functional selection for catalytic activity.

### EP-Seq expression scores combined with sequence conservation reveal functional sites in DAOx

**Fig. 4:**
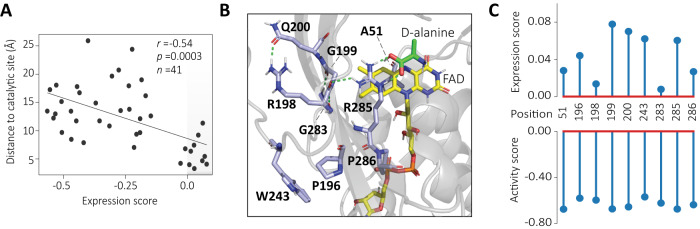
EP-Seq and sequence conservation analysis reveal functional sites of DAOx. **A** The distance to the catalytic site for positions that are evolutionarily conserved (conservation score, CONS = 100) were plotted as a function of EP-Seq expression scores (average per position) and analyzed through Pearson linear correlation function with a two-sided statistical test. The nine highly conserved residues associated with the most positive expression scores (light gray right corner) are visualized in (**B**). **B** Structural details of DAOx residues with CONS = 100 and expression score > 0. H-bonds are shown in green. The FAD cofactor and D-alanine carbon backbones are colored in yellow and green, respectively. DAOx residue carbon backbones are colored in light blue with colored elements: Hydrogen (white), Nitrogen (dark blue), Oxygen (red), Phosphorus (orange). **C** Expression and activity scores for each of the conserved positions linked to positive expression scores.

Sequence conservation scores derived from multisequence alignment reflect constraints imposed on the protein of interest as a result of natural selection. Over evolutionary time, selection tends to maintain both folding stability and catalytic activity^60,61^. We hypothesized that combining conservation scores with our stability and activity datasets could provide insights into functional sites in DAOx. We compared the experimental scores with conservation scores (CONS) obtained by aligning the DAOx sequence with five evolutionarily related D-amino acid oxidase sequences^62^ (Supplementary Fig. 7). Both expression and activity fitness score datasets showed negative correlation with conservation scores (Exp *r* = −0.29, *p* < 0.0001; Act *r* = −0.40, *p* < 0.0001, Fig. 3C, Supplementary Fig. 6F), generally indicating higher tolerance for mutations at less conserved protein sites. We identified 41 positions with CONS = 100 and analyzed the average expression score per position as a function of the distance of the mutated site from the catalytic center of DAOx. We observed a significant negative correlation of the expression scores with the distance of the mutation from the catalytic site of the enzyme (*r* = −0.54, *p* = 0.0003, Fig. 4A). Among the sites with conservation score 100, we then isolated the positions associated with positive expression score (*n* = 9) and visualized them on the 3D structure of DAOx (Fig. 4B). Three of the positions analyzed (A51, G199, R285) found within 5 Å of the active site are reported to be directly involved in catalysis through stabilization of the substrate D-alanine (R285) or of the isoalloxazine ring of FAD cofactor (A51, G199)^35^. Conserved residues R198, G199, Q200, G283, and the two prolines in position 196 and 286, are part of two antiparallel strands β8 and β13 suggesting a role of the resulting β sheet in structuring the catalytic pocket and coordinating the side chain of the arginine 285. Finally, tryptophan 243 located at the surface of monomeric DAOx plays an important role in the dimerization. In agreement with the previous observations, its mutation has a direct impact on the catalytic efficiency of the DAOx^63^ (Fig. 4C).

### Activity enhancing hotspots are globally encoded

**Fig. 5:**
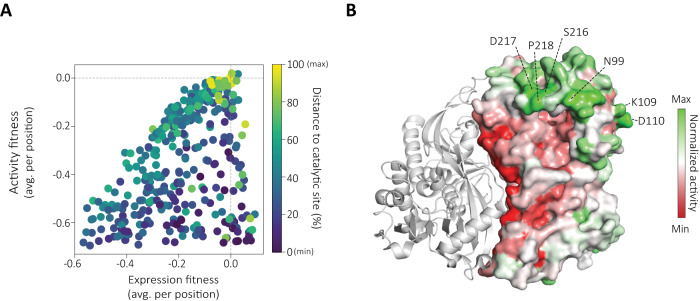
EP-Seq predicts activity enhancing mutations in DAOx. **A** Average expression and activity fitness scores per position in the DAOx enzyme. The coloring indicates relative distance to the active site. **B** Normalized activity values are depicted on the 3D structure of a DAOx monomer (right). Areas of the enzyme where mutations on average led to increased activity are represented in green, while regions where mutations on average had a negative impact on activity are shown in red. The most frequent 6 positions among the top 1000 mutants with highest normalized activity scores are indicated.

We finally used EP-Seq to identify regions of the enzyme where mutations were associated with improved catalytic activity. We generated a two-dimensional scatterplot showing the average activity and expression fitness per position as Y and X coordinates (Fig. 5A), and assigned a color at each point based on the distance of the mutated site to the enzyme’s catalytic pocket. The observed trends in this depiction demonstrate how the two properties are related. Variants that are found to be catalytically active must possess at least a minimum level of stability in order to be correctly folded, secreted and displayed. This fact is demonstrated by a lack of positions in the upper left of the plot. Only 1 datapoint (~0.3%) was found with positive activity fitness and negative expression fitness (Fig. 5A, top left). Additionally, we found 35 positions (~10%) exhibiting positive expression fitness with impaired catalytic activity (activity fitness < −0.1) (Fig. 5A, lower right). These positions tended on average to be closer to the catalytic site (avg. distance = 14.6 Å; avg. distance all = 17.4 Å), indicated by the dark blue color in the lower right of the plot. This demonstrates stability-function tradeoffs at play in this enzyme, where more stably expressed variants could be found by mutating crucial catalytic residues at the cost of losing activity. Finally, many mutations which moderately altered the expression of the enzyme resulted in corresponding changes in the catalytic activity (Fig. 5A, diagonal). A limited number of positions were tolerant to mutations for both assayed properties (*n* = 23) (Exp > −0.05; Act > −0.05) and showed either simultaneous improvement of both properties or an increase in activity due to higher expression levels on the surface of the yeast cells. These tended to be found more distant from the catalytic site (avg. distance 28.2 Å; avg. distance all = 17.4 Å). To further support these observations, we have included a scatter plot to illustrate the relationship between activity and expression fitness for all the single mutant variants included in our work (Supplementary Fig. 8A).

We finally deconvoluted the activity score from the expression score in order to identify regions of the enzyme susceptible to catalytic improvement. We first selected all single mutant variants with expression fitness values higher than the upper limit observed for nonsense mutants (Expression fitness = −0.69 ± 0.04; Fig. 2F). We then normalized the non-logarithmic activity fitness of 6362 single mutants relative to their respective expression fitness values. These normalized activity scores exhibited reduced sensitivity to mutations’ impact on enzyme folding stability, as evidenced by the decrease in correlation with FOLDX algorithm predicted values, reduced from rho = −0.59 (*n* = 354, *p* < 0.0001, Supplementary Fig. 4D) to rho = 0.3 (*n* = 354, *p* < 0.0001). Figure 5B shows the enzyme structure colored according to normalized activity. This normalization process resulted in 2029 variants with normalized activity scores exceeding 1, indicating enhancements in catalytic activity independent of expression levels (Supplementary Fig. 8B, C). We then ranked all variants based on their normalized activity scores and selected the top 1000 variants from the entire dataset. We extracted the six most frequently represented amino acid positions within this subset and indicated them on the three-dimensional structure of the DAOx (Fig. 5B). Four out of the six identified positions were found to be closely situated to the substrate tunnel region, through which D-alanine approaches the enzyme and enters the active site (positions: 99, 216, 217, 218; Fig. 5B). In particular, S216, D217, and P218 are components of the loop positioned at the entrance of the tunnel and are in close proximity to the asparagine in position 99. These findings suggest that this region plays a role in controlling the accessibility of the substrate tunnel, through interactions with the substrate itself or by dynamically reshaping the enzyme’s structure to facilitate the entry of D-alanine into the reaction site. The other two positions identified, K109 and D110, are located on the opposite side of the dimer interface. Modifying these positions could potentially trigger conformational changes in the overall enzyme structure, which in turn may lead to allosteric effects on the enzyme’s catalytic function (Fig. 5B).

## Discussion

EP-Seq is a deep mutational scanning workflow for studying enzyme folding stability and catalytic activity. Yeast surface display combined with peroxidase-mediated proximity labeling of single cells was able to link enzymatic activity to fluorescent phenotypes for large libraries in a pooled format, enabling cell sorting and high-throughput sequencing. We demonstrated our workflow by constructing a near comprehensive single substitution variant library of DAOx, and assaying it by EP-Seq for folding stability and catalytic activity. By jointly analyzing the expression and activity fitness datasets as a function of various biophysical and biochemical properties of the mutated residues at WT residues, we gained structural and biophysical insight into DAOx. When considering the correlation of fitness score with various biophysical parameters of the mutated residues, for many parameters the two datasets exhibited high concordance. This shows how in order for a variant to be catalytically active, it must first be stably expressed and secreted to the cell wall. We therefore observed an expression level-dependency of the catalytic activity. However, our data further revealed certain properties of mutated sites which were differentially correlated with expression and activity fitness. These included distance of the mutated residue to the FAD cofactor and distance to the dimer interface. We found that WT residues in close proximity to the FAD cofactor and in the active site tended to destabilize the enzyme. These residues could be mutated to enhance folding stability, primarily through hydrophobic effects (Fig. 3D i and Supplementary Fig. 8D). However, this increased stability was mostly achieved at the cost of catalytic activity (see Fig. 3D iv and Supplementary Fig. 8E). These observations suggest that functional constraints have contributed to shape the evolution of DAOx over time, and provide direct and clear evidence of an activity-stability tradeoff. The distance of the mutated residues to the dimer interface was similarly decoupled between expression and activity fitness datasets. Prior literature^58,59^ indicates dimerization is necessary but not sufficient for catalytic activity. Mutations that disrupted the dimer interface therefore functioned similarly to those in the active site, where destabilizing the dimer interface through mutation could lead to higher overall expression, however this increased stability was achieved at the cost of catalytic activity. Our workflow is compatible with enzymes that can be functionally displayed on the cell surface and whose activity can be directly or indirectly (via enzymatic cascade) linked to the production of peroxide. This includes enzymes with immediate therapeutic relevance such as Arginase^64^, and Asparaginase (Supplementary Fig. 9), as well as biocatalysts with diagnostic or industrial applications, like Glucose Oxidase^28^. Due to its inherent scalability, our approach will find applications in generating training data for machine learning algorithms, which represents a major challenge for catalytic enzymes. In the future, EP-Seq can contribute to a better understanding of evolutionary processes in natural enzymes, help in identifying functional allosteric sites, and be used to evolve protein catalysts for industrial and biomedical applications.

## Methods

### DAOx sequence cloning

The gene coding for *Rhodotorula gracilis* D-amino acid oxidase (DAOx) was acquired from Twist Bioscience codon optimized for expression in *Saccharomyces cerevisiae*. The sequence was cloned after restriction digestion with BamHI and XhoI into a pYDKan plasmid, a modified pYD1 yeast display vector where the original glycine-serine linker between Aga2 and the protein of interest was replaced with the protein linker GTPTPTPTPTGEF^65^ and the β-lactamase gene replaced with a kanamycin resistance gene. After confirming the sequence through Sanger sequencing the plasmid pYDKan_RgDAOx was transformed through a lithium acetate transformation protocol^66^ in the yeast strain EBY100 and the positive colonies selected on synthetic defined (SD) agar 2% (wt/vol) glucose plates lacking tryptophan (-Trp). We provide the sequence of the pYDKan_wtRgDAOx here: https://zenodo.org/record/8388902.

### Expression and surface display of DAOx wild type

Positive transformants were cultivated in -Trp liquid medium with 2% (wt/vol) glucose for 24 h at 30 °C to an OD_600_ ~ 8 with continuous shaking at 200 rpm. Expression and display of the Aga2_DAOx fusion protein were tested by transferring the cells to fresh -Trp liquid medium with 0.2% (wt/vol) glucose and 1.8% (wt/vol) galactose further supplemented with 100 mM citrate/phosphate buffer at pH7. In the optimization step of the protocol cells were transferred in an induction medium without buffer or with 100 mM citrate/phosphate buffer at pH range 3–7. Yeast cells were grown in induction media for 48 h at 20 °C before being pelleted, washed with PBS 0.1% (wt/vol) bovine serum albumin (BSA) and used for antibody labeling in order to detect the c-terminal 6xHistidine (6xHis) tag of the displayed fusion protein.

### Yeast antibody staining to detect surface displayed DAOx

After induction of protein expression and display, yeast cells were washed twice with PBS 0.1% (wt/vol) BSA before being resuspended at a concentration of 2 million cells/100 μl in the same buffer with the addition of 1/500 dilution of anti-6x-His mouse monoclonal antibodies (Thermo Fisher Scientific Cat# MA1-21315, RRID: AB_557403). The samples were incubated with the primary antibody 30 min at room temperature before being washed twice with PBS 0.1% (wt/vol) BSA. After, the cells were resuspended at the concentration of 2 million cells/100 μl in PBS 0.1% (wt/vol) BSA with 1/500 dilution of a secondary goat anti-mouse antibody conjugated with Alexa Fluor™ 594 (Thermo Fisher Scientific Cat#A-11005, RRID: AB_2534073). The samples were incubated with the secondary antibody for 30 min at 4 °C before being washed twice with PBS 0.1% (wt/vol) BSA and analyzed through flow cytometry. Surface expression of DAOx was determined through flow cytometry using a Attune NxT (Thermo Fisher Scientific) cytometer equipped with a 488 nm and a 561 nm laser. For flow cytometry, 10,000 cells per sample were recorded and analyzed.

### DAOx Amplex Red activity assay

Yeast cell populations positive for the display of DAOx were assayed through Amplex Red activity assay to detect D-amino acid oxidase activity. Half a million yeast cells were mixed together with 35 mM D-alanine, 5.6 μM HRP, and 100 μM Amplex Red in PBS (pH 7.5). The fluorescence was read at 590 nm.

### DAOx mutant library construction and barcoding

Mutagenesis of the DAOx wild type gene was performed through a plasmid based one-pot nicking mutagenesis protocol^67^. The method involves successive nicking, degradation, and de novo synthesis of plasmid DNA strands. Nt.BbvCI and Nb.BbvCI endonucleases identify the same BbvCI site on the target dsDNA plasmid but nick only one strand. After incubating the target plasmid DNA with one of these endonucleases and then with an exonuclease for degradation, a new DNA strand is synthesized through thermal cycling. This process uses 5’ phosphorylated NNK oligos and a high-fidelity DNA polymerase, extending the primer around the circular template. A low primer to template ratio favors the annealing of just one primer per template, leading to the synthesis of primarily single mutant variants. Finally, Taq DNA ligase closes the new strand, creating a dsDNA plasmid with a mismatch at the mutation site. Repeating the process on the other DNA strand of the template using the other nicking endonuclease and a non-mutagenic phosphorylated oligo in the amplification step targeting a constant plasmid region, completes the synthesis of dsDNA variant plasmids. We first transferred the entire DAOx expression cassette, inclusive of galactose promoter, Aga2- WT DAOx open reading frame (ORF) and Matα transcription terminator from pYDKan (5498 bp) to a smaller pUC19 plasmid (4265 bp). The new plasmid was further engineered through the insertion of two BbvCI restriction sites located upstream and downstream of the expression cassette. The original nicking mutagenesis protocol^67^ was integrated with an additional step: after the first incubation with Nt.BbvCI enzyme and exonucleases, the reaction mixture was further incubated with 10 units of Quick CIP phosphatase (New England Biolabs) at 37 °C for 20 min, followed by incubation at 80 °C for 20 min. This step promotes the removal of 5’ phosphate from nicked and partially degraded strands of wild type DNA and prevents reformation of closed double strand wild type DNA plasmid in the following amplification and ligation steps. For the mutagenic DNA amplification step of the protocol we used nested NNK forward primers targeting codons 2 to 365 of the DAOx wild type sequence. Primers were designed using the script create_primers.py^68^. The length of each primer was adjusted within 25 and 51 nucleotides in order to obtain oligonucleotides with similar melting temperature in the range of 60–61 °C. We ordered hand-mixed degenerate oligonucleotides in 96 well plate form from Integrated DNA technologies and mixed the primers in equimolar proportion at a final concentration of 10 μM. Finally, the one pot site saturation mutagenesis protocol was performed into two subsequent cycles. The pool of plasmids recovered after the first cycle was used as a template for the second cycle. Equimolar amounts of the resulting plasmids from the two rounds of mutagenesis were merged composing the final mutant library. Unique molecular identifier (15 N) DNA barcodes were linked through standard PCR to each of the DAOx mutagenized inserts by using primers F1 and R1 (Supplementary Table 3). Nucleotide barcodes were added to the 5’ end of the amplicon, upstream of the galactose promoter. Amplified and barcoded expression cassettes with mutagenized DAOx coding sequences were cloned into a BbVCI/PmeI digested pYDKan vector using HiFi DNA Assembly (New England Biolabs). Assembled products were column purified (Zymo-Spin I, Zymo Research) before being electroporated into XL1-Blue electroporation competent cells (Agilent, 200228). Positive colonies were selected on 15 cm LB agar plates containing kanamycin (50 μg/ml). The final barcoded library size was capped through single cell sorting to 200,000 cells. After sorting, cells were incubated for 20 h and then used to extract plasmid DNA. 5 μg of the library plasmid pool was transformed into the final yeast *Saccharomyces cerevisiae* strain EBY100 through lithium acetate transformation^66^. Serial dilutions of the final transformation reactions were plated on -Trp agar plates with 2% (wt/vol) glucose. Quantification of the colonies forming units from the dilution plates indicated a final transformation yield between 6 and 10 M colonies. After transformation, the yeast mutant library was grown for 24 h at 30 °C in -Trp liquid medium supplemented with 2% (wt/vol) glucose before being diluted to OD_600_ = 1 into fresh -Trp medium supplemented with 2% (wt/vol) glucose and grown for further 16 h at 30 °C. Finally, aliquots of 10^8^ cells were prepared and resuspended in 25% (vol/vol) glycerol before being stored at −80 °C. Sequences of pUC19_RgDAOx plasmid and mutagenic primers are provided here: https://zenodo.org/record/8388902.

### Pacific Biosciences long read sequencing and data analysis

We used PacBio long read sequencing to establish the link between the DAOx variants represented in our mutant library and the 15 nucleotides unique molecular identifiers (UMI). We sequenced the mutant library before transformation of the mutagenized and barcoded pool of plasmids into the final yeast strain EBY100. PacBio sequencing was performed on bacterial extracted plasmid DNA. This procedure eliminates the need of preparing the sequencing insert via PCR and thus the possibility of strand exchange events. Barcoded plasmids were purified from bacterial cells and the sequencing inserts were prepared through restriction digestion with PmeI and NotI restriction enzymes. The final dsDNA pool was purified after agarose gel electrophoresis and used for SMRTbell ligation. PacBio sequencing run was performed on a Sequel IIe system with a 30 h movie collection time using a SMRT cell 8 M. Final PacBio circular consensus sequences (CCSs) were generated using the ccs program from Pacific Bioscience (https://github.com/PacificBiosciences/ccs) and filtered in order to retain DNA sequences with average Phred quality score higher than Q20. The final sequencing file is available at https://zenodo.org/record/8388902.

We established a computational workflow to map, extract and link each UMI represented in our library to the respective DAOx variant sequence. Through Minimap2^69^ (version 2.19), using a reference sequence (DAOx_ref.fa), we mapped on each read the region corresponding to the UMI and the mutations falling in the Aga2-DAOx open reading frame.

$${{{{{\rm{minimap}}}}}}2-{{{{{\rm{cs}}}}}}-{{{{{\rm{ax}}}}}}\; {{{{{\rm{map}}}}}}-{{{{{\rm{hifi}}}}}}\; {{{{{\rm{DAOx}}}}}}\_{{{{{\rm{ref}}}}}}.{{{{{\rm{fa\; pacbio}}}}}}.{{{{{\rm{fastq}}}}}} > {{{{{\rm{aln}}}}}}.{{{{{\rm{sam}}}}}}$$

The output of Minimap2 alignment was processed further by using a C program called ppba.c. This program was specifically designed for this task and it makes use of a custom library file named libsccodon.h. Information regarding the nucleotide sequence of each barcode, barcodes sequencing quality per position, mutations falling in the Aga2-DAOx ORF and presence of indels were extracted and stored into a first version of the look-up table (pre_lut).

$$./{{{{{\rm{ppba}}}}}}\; {{{{{\rm{DAOx}}}}}}\_{{{{{\rm{ref}}}}}}.{{{{{\rm{fa}}}}}}\; {{{{{\rm{aln}}}}}}.{{{{{\rm{sam}}}}}} \, > \, {{{{{\rm{pre}}}}}}\_{{{{{\rm{lut}}}}}}.{{{{{\rm{tsv}}}}}}$$

The resulting look up table file was processed to remove conflicting nucleotide barcodes through the python notebook Generate_Lut.ipynb. Identical UMIs associated with different DAOx variant sequences were compared. Among those, we retained in the final version of the look-up table UMIs whose link to a specific mutation was confirmed by more independent sequencing reads or UMIs with higher sequencing quality and linked to lower number of indels in the sequencing read. If none of these criteria could be used to classify the UMIs, conflicting barcodes were all removed from the final look-up table. UMIs linked to mutations registered out of the targeted region (codon 2-365 of DAOx) were kept in the lookup table and filtered out in a later stage of the analysis. The resulting look-up table file was further processed in order to finalize the table and remove all the nucleotide barcodes with length different than 15 nucleotides. The C programs used for this analysis were compiled by using GCC (version 7.4.0) on a 32-bit processor. The scripts, sequencing files and the final look-up table are available at https://zenodo.org/record/8388902.

### DAOx variant library expression and display

100 M yeast cells transformed with the barcoded DAOx variant library were inoculated at starting OD_600_ = 0.1 in 100 ml of -Trp liquid medium with 2% (wt/vol) glucose and grown for 24 h at 30 °C to an OD_600_ ~ 8 with continuous shaking at 200 rpm. Protein expression and display were induced by transferring the yeast cells at a starting OD_600_ of 0.4 to a 100 ml fresh -Trp liquid culture with 0.2% (wt/vol) glucose and 1.8% (wt/vol) galactose. The induction medium was further supplemented with 100 mM citrate-phosphate buffer at pH7. Cells were cultivated in the induction medium for 48 h at 20 °C shaking at 200 rpm, before being washed twice with 25 ml PBS 0.1% (wt/vol) BSA. 120 M induced cells were distributed into 6 tubes at a concentration of 20 M cells/ml. Detection of the displayed Aga2-DAOx protein construct was performed in PBS 0.1% (wt/vol) BSA incubating the cells with 1/500 dilution of the anti 6xHis-tag mouse monoclonal antibodies (Thermo Fisher Scientific Cat# MA1-21315, RRID: AB_557403) for 30 min at room temperature. After incubation with the primary antibody, cells were washed twice with ice cold PBS 0.1% (wt/vol) BSA buffer before being resuspended in the same buffer with 1/500 dilution of a secondary goat anti-mouse antibody conjugated with Alexa Fluor™ 594 (Thermo Fisher Scientific Cat#A-11005, RRID: AB_2534073). The reaction was then incubated at 4 °C for 30 min. After the incubation the cells were washed twice and resuspended in ice-cold PBS 0.1% (wt/vol) BSA at a concentration of 20 M cells/ml. In parallel, a negative control staining procedure was performed. In a single tube 20 M induced yeast cells were incubated exclusively with the secondary goat anti-mouse antibody conjugated with Alexa Fluor™ 594 (Thermo Fisher Scientific Cat#A-11005, RRID: AB_2534073) at the same conditions described above. This sample was then used to detect eventual fluorescence signals caused by unspecific binding of the fluorescent secondary antibody to the yeast cells.

### DAOx single cell tyramide/peroxidase proximity labeling assay

Catalytic activity of displayed DAOx wild type and variant enzymes was assayed through a single-cell tyramide/peroxidase proximity labeling method. After induction of protein expression and cell surface staining of the yeast population as described above, 25 M yeast cells were mixed at a concentration of 500 cells/μl with 1/200 dilution of Alexa fluor™ 488 Tyramide Reagent (Thermo Fisher, B40953), 56.8 μM HRP (Sigma-Aldrich, 77332) and 130 mM D-alanine in 1 × PBS and 0.75% (w/v) sodium alginate. The reaction was incubated for 20 min at 25 °C. Afterwards, the cells were spun down for 3 min at 13000 g in a table top centrifuge. The cell pellet was washed twice with PBS 0.1% (wt/vol) BSA + 0.05% (vol/vol) Tween 20 and then used for flow cytometry and single cell sorting experiments.

### Fluorescence activated cell sorting (FACS) of the DAOx yeast library

Expression level sorting: yeast cells stained for the expression and display of DAOx variants were sorted using a FACSMelody cell sorter equipped with 488 and 561 nm lasers and a 100 μm nozzle. Cells were sorted into pre-wet 5 ml FACS tubes containing 0.5 ml of 2X -Trp medium with 4% (wt/vol) glucose and 1% (wt/vol) BSA. Yeasts were first gated for single events and the population further divided into 4 sorting bins along the Alexa Fluor™ 594 fluorescence axis. The first bin (Gate1, Fig. 2A) was set in order to capture the 99% of non-fluorescent cells by using the negative control sample of the antibody staining procedure as reference. The remaining part of the cell population was divided into three further sorting bins capturing each an equivalent fraction of the yeast population with increasing fluorescence intensity (Gates 2, 3, 4, Fig. 2A). The sorting procedure was repeated two independent times sorting each time more than 10 M single yeast cells (Supplementary Table 2).

Activity level sorting: EBY100 yeast cells stained for the expression of the DAOx enzyme variants and assayed through single cell tyramide activity assay were sorted using a FACSMelody cell sorter equipped with 488 and 561 nm lasers and a 100 μm nozzle. As above, cells were sorted into pre-wet 5 ml FACS tubes containing 0.5 ml of 2X -Trp medium with 4% (wt/vol) glucose and 1% (wt/vol) BSA. Yeast cells were first gated for singleton events and then the population divided into four bins based on the level of green fluorescent signal. Bin 1 was designed in order to include 99% of the population of cells negative to display or displaying inactive DAOx variants, using as reference the fluorescence level of the non-displaying yeast populations (Gate 1, Fig. 2C). The remaining part of the population of cells was equally divided into three yeast sub-populations with increasing fluorescent signal (Gates 2, 3, 4, Fig. 2C). We performed the sorting of two independently assayed yeast populations sorting each time more than 8 M total yeast cells (Supplementary Table 2).

After each cycle of sorting both for expression and activity, yeast cells part of the same gated population were merged into 50 ml falcon tubes and pelleted 10 min at 4000 g in a table top centrifuge. Afterwards, the supernatant was discarded and the cell pellet resuspended in 10 ml -Trp medium with 2% glucose supplemented with 100 μg/ml Penstrep. After sorting, all the cell populations were grown for 48 h at 30 °C shaking at 200 rpm before being sampled into aliquots of 50 M cells each and stored at −80 °C in 25% (v/v) glycerol.

### DNA prep for Illumina sequencing

50 M yeast cells per sorted population were used as starting material for the preparation of Illumina sequencing inserts. Cells were first collected from −80 °C and incubated 5 min at RT before being spun down 1 min at 13,000 g in a table top centrifuge. The supernatant was discarded and the cell pellet resuspended in 250 μl of miniprep resuspension solution (GeneJET Plasmid Miniprep Kit, Thermo Fisher) with the addition of 20 U of Zymolyase (Zymo Research). The reaction was incubated for 2 h at 37 °C shaking at 900 rpm. After this incubation step the samples were processed following a typical plasmid miniprep kit protocol (GeneJET Plasmid Miniprep Kit, Thermo Fisher). Finally, plasmid DNA extracted from yeast cells was eluted in 15 µl of nuclease free water. The region of the plasmids containing the 15 N UMI was amplified through a standard PCR reaction using the NEBNext Ultra II Q5 Master Mix (New England Biolabs). 25 µl of the master mix were mixed with 5 µl of each primer (1 µM) and 15 µl of template DNA. Primers were designed accordingly in order to target the region of interest and be compatible with the Nextera indexing library preparation. An equimolar mixture of four staggered primers was used in each sample preparation as forward and reverse primer in order to provide in both ends of the resulting amplicon different starting nucleotides for the Illumina reads (F3-6 and R3-6 primers in Supplementary Table 3). PCR was performed with the following program: 1 cycle at 98 °C for 30 s, 18 cycles at 98 °C for 10 s, 72 °C for 30 s, 72 °C for 2 min and a final elongation step of 2’ at 72 °C before storing the reaction at 4 °C. Resulting DNA amplicons were then visualized through DNA electrophoresis on a 1% (wt/vol) agarose gel in and purified from the gel using a standard DNA purification kit. The concentration of DNA per sample was measured and DNA was purified once more through the DNA Clean and Concentrator-5 kit (Zymo Research). After addition of unique Nextera indexing sequences the samples were pooled and sequenced through Novaseq 6000 Illumina sequencing. Numbers of Illumina reads per sample are provided in Supplementary Table 2.

Demultiplexed reads were then processed through a computational pipeline in order to extract the sequences of UMIs and align them to the information contained in the look-up table.

Sequences corresponding to the UMI were first mapped through BBMap alignment algorithm^70^ by using a reference sequence file (ill_ref.fa)

$${{{{{\rm{bbmap}}}}}}.{{{{{\rm{sh}}}}}}\; {{{{{\rm{in}}}}}}=\ast .{{{{{\rm{fastq}}}}}}\; {{{{{\rm{ref}}}}}}={{{{{\rm{ill}}}}}}\_{{{{{\rm{ref}}}}}}.{{{{{\rm{fa}}}}}}\; {{{{{\rm{out}}}}}}=\ast .{{{{{\rm{sam}}}}}}$$

BBMap *.sam outputs were further processed through the C script pib.c (process illumina barcodes). Through this step we extracted the sequences and read quality of each UMI mapped by BBMap, saving the information in a fastq format file.

$$./{{{{{\rm{pib}}}}}}*.{{{{{\rm{sam}}}}}} > *.{{{{{\rm{fq}}}}}}$$

Each UMI mapped and registered was then aligned to the information stored in the look-up table through the C script rib.c (read illumina barcodes). The following tags: 0 - not found in the look-up table, 1 - found in the look-up table, 2 - read quality <Q20, 3 - UMI size different than 15 N, were associated with each of the barcodes read through Illumina sequencing. We applied a quality filter of Q20, therefore tagging with tag 2 all the UMI associated with a read quality lower than Q20.

$$./{{{{{\rm{rib}}}}}}-{{{{{\rm{t}}}}}}\; {{{{{\rm{lut}}}}}}\_{{{{{\rm{m}}}}}}.{{{{{\rm{tsv}}}}}}-{{{{{\rm{q}}}}}}20*.{{{{{\rm{fq}}}}}} \, > \,*.{{{{{\rm{tsv}}}}}}$$

Finally, UMI sequences with tag 1 (found in the look-up table), were extracted, sorted alphabetically and grouped by identity.

$${{{{{{\rm{grep}}}}}}}^{1}\ast .{{{{{\rm{tsv}}}}}}|{{{{{\rm{sort}}}}}}|{{{{{\rm{uniq}}}}}}-{{{{{\rm{c}}}}}}|{{{{{\rm{sed}}}}}}-{{{{{\rm{E}}}}}}^{\prime} {{{{{\rm{s}}}}}}{/}{\ast }// ; s // \backslash t/^{\prime} { > {{{{{\rm{t1sct}}}}}}}{\ast }.{{{{{\rm{tsv}}}}}}$$

All the Illumina reads files were processed through the reported computational workflow. Finally, all the information about the UMI sequences and count found in each bin (t1sct_Bin#.tsv) were merged in a unique file using the python notebook ill_tag1_bins.

The C programs used for this analysis were compiled by using GCC (version 7.4.0) on a 32-bit processor. The scripts and sequencing files are available at https://zenodo.org/record/8388902.

### Expression and activity fitness scores calculation

The number of sequencing reads linked to each variant enzyme (*r*_v_) was converted into number of sorted cells of the same variant (*c*_*v*_) per sorted bin using the following Eq. 1:

1 $$\frac{{r}_{v}}{{r}_{{tot}}}=\frac{{c}_{v}}{{c}_{{tot}}}$$

where *r*_*tot*_ is the total number of illumina reads of the bin and *c*_*tot*_ is the total number of cells sorted in the same bin. Then, the final expression and activity scores per variant were computed as the expected value of fluorescent intensity of the variant across all the four bins of the experiment. We calculated first a weighted mean (*β*) of the cell numbers (*c*_*v*_) where the median fluorescence of the yeast population in each bin (*ω*) was used as the weighting factor:

2 $$\beta=\frac{{\sum }_{i=1}^{{bin}}{\omega }_{i}\cdot {c}_{{vi}}}{{\sum }_{i=1}^{{bin}}{c}_{{vi}}}$$

The expression and activity fitness score (*F*) per variant were finally calculated as follow:

3 $$F=lo{g}_{2}\left(\frac{{\beta }_{v}}{{\beta }_{wt}}\right)$$

where ${\beta }_{v}$ is the weighted mean expression score of the variant enzyme and ${\beta }_{{wt}}$ is the score of wild type DAOx. To calculate the final consensus fitness score (*F*_*fin*_) for each variant enzyme, we used a weighted mean of the single fitness scores recorded in each replicate (*F*_*v*_) of both the expression and activity assays. The weighting factor was the number of cells associated with the measured fitness in each experiment (*c*_*v*_) (Eq. 4).

4 $${F}_{fin}=\frac{{\sum }_{j=1}^{rep}{F}_{vj\cdot {c}_{vj}}}{{\sum }_{j=1}^{rep}{c}_{vj}}$$

### FoldX ΔΔG calculation

In order to predict the effect of missense mutations on the stability of the DAOx monomeric structure we used the FOLDX algorithm^52^ (version 5.0). We first processed the DAOx crystal structure (PDB: 1C0P) through the FOLDX RepairPDB function in order to identify and repair residues of the structure with bad torsion angles or energy clashes. The repaired structure was then processed through the FOLDX tool PositionScan that mutates the residue of the structure (positions 2-361) to the 20 natural amino acids providing an estimation of the change in energy between the folded and unfolded state of the wild type protein and comparing it to the change in folding energy upon amino acid mutation. The final score ΔΔG was then calculated through the following equation:

5 $$\Delta \Delta {{{{{\rm{G}}}}}}=\Delta {{{{{\rm{GMut}}}}}}-\Delta {{{{{\rm{GWt}}}}}}$$

Positive values of predicted ΔΔG scores indicate a negative effect of mutations on the stability of the structure. Negative values of ΔΔG predictions indicate positive effect of mutations on the overall stability of the structure. A mean ΔΔG score per position of the protein was calculated and the final values (*n* = 360) were correlated to experimental expression and activity fitness scores. Input-output and setting files used for the ΔΔG prediction are provided at https://zenodo.org/record/8388902.

### DAOx structural properties calculation and extraction

Solvent accessible surface area (SASA) was used as a measure of residue solvent exposure. SASA scores were calculated per residue of the DAOx wild type monomeric protein (PDB: 1C0P) through the PyMol tool get_sasa_relative (https://pymolwiki.org/index.php/Get_sasa_relative). Scores ranging between 0 (minimum solvent exposure) and 100 (maximum solvent exposure) were extracted and analyzed in relation to the activity and expression fitness scores. B-factor scores as a scale of thermal induced dynamic disorder and flexibility of the structure were extracted per position of DAOx monomeric structure (PDB file 1C0P.pdb). Residues involved in the monomer-monomer contact at the dimer interface of DAOx dimeric structure (PDB:1C0P) were selected through the pyMol tool InterfaceResidues (https://pymolwiki.org/index.php/InterfaceResidues).Coordinates of the alpha carbons (Cα) of interface residues were extracted from the DAOx structure file (PDB:1C0P) and the distance between each residue of the structure to the closest interface Cα was calculated. Spatial coordinates of all the atoms of the flavin adenine dinucleotide (FAD) cofactor were extracted from the 3D structure of DAOx wild type (PDB:1C0P). We calculated the distance of each Cα of DAOx monomeric protein to each of the FAD cofactors atoms. A final mean value distance per residue to the FAD was calculated by computing the arithmetic mean of all the distances of the same residue to the FAD atoms.

### Single mutant variants expression and activity level

From the 6399 single mutant variants of DAOx analyzed through our DMS workflow both for expression and activity, we randomly selected 12 single mutant DAOx variants: S48C, L153R, A187K, A187E, G199E, Q200W, S215A, T237M, S268Y, P292E, L310P, G315P. Genes coding for each of the selected DAOx variants and codon optimized for the expression in *Saccharomyces cerevisiae* were synthesized and cloned into a recipient pYDKan plasmid in frame with Aga2p coding sequence on a BioXP 3250 synthetic biology workstation (Codex DNA). All the sequences were confirmed through Sanger sequencing and the final plasmids were transformed into the yeast strain EBY100 through lithium acetate transformation protocol. Positive colonies were selected on synthetic defined (SD) agar 2% (wt/vol) glucose plates lacking tryptophan (-Trp). Positive colonies were cultivated in -Trp liquid medium with 2% (wt/vol) Glucose for 24 h at 30 °C to an OD_600_ ~ 8 with continuous shaking at 200 rpm. Expression and display of the Aga2-DAOx wild type and mutant constructs were induced by transferring the cells at OD_600_ = 0.4 to fresh -Trp liquid medium with 0.2% (wt/vol) glucose and 1.8% (wt/vol) galactose and supplemented with 100 mM citrate/phosphate buffer at pH7. The cells were grown in induction medium for 48 h at 20 °C before being pelleted, washed with PBS 0.1% (wt/vol) BSA and used for antibody labeling in order to detect the c-terminal 6xHistidine (6xHis) tag of the displayed fusion protein.

Yeast cells stained for the expression of DAOx wild type and mutant variants were analyzed through flow cytometry. Yeasts were first gated for single events and the resulting population of cells was further divided into 4 gates along the Alexa Fluor™ 594 fluorescence axis. Gate 1, set between fluorescence values 0-2500, included the 99% of the non-stained yeast population used as negative control. Other 3 gates were set respectively at 2500-25000, 25000-250000, 250000-2500000 fluorescence arbitrary units in order to cover the entire range of fluorescence. 10’000 single cells from each yeast population were analyzed. We recorded the median value of the population in each of the four gates and the % of yeast cells represented in each gate. Each population was assayed for expression three independent times. We calculated a weighted mean (*β*) of the distribution of cells (%*c*) where the median fluorescence of the yeast population in each gate (*ω*) was used as the weighting factor (Eq. 6).

6 $$\beta=\frac{{\sum }_{i=1}^{{{{{{\rm{gate}}}}}}}{\omega }_{i}\cdot\%{c}_{i}}{{\sum }_{i=1}^{{{{{{\rm{gate}}}}}}}\%{c}_{i}}$$

Finally, the single clone expression score (*F*_*sing. clone*_) was calculated by dividing the weighted mean expression of each DAOx variant tested (*β*_*v*_) by the weighted mean of the wild type DAOx (*β*_*wt*_) assayed through the same procedure (Eq. 7)

7 $${F}_{{sing}.{clone}}=\frac{{\beta }_{v}}{{\beta }_{{wt}}}$$

To measure the activity of DAOx wild type and mutant variants, yeast cell populations expressing these variants were assayed using the Amplex Red method with D-alanine as the substrate. A mixture of half a million yeast cells, 35 mM D-alanine, 5.6 μM HRP, and 100 μM Amplex Red in PBS (pH 7.5) was prepared. The fluorescence signal was then measured at 590 nm every 60 s for a total of 30 min. The linear range of the reaction for all the variant enzymes was determined between 0 and 10 min of incubation, and the slope of each reaction was calculated. The single clone activity score for each variant was obtained by dividing the slope of the reaction for the variant by the slope of the reaction for the wild type enzyme. The final scores were calculated as the average of six independent measurements per cell population.

### Reporting summary

Further information on research design is available in the Nature Portfolio Reporting Summary linked to this article.

### Supplementary information

Supplementary Information

Peer Review File

Reporting Summary

## Data Availability

All the data required for replicating the study, DNA sequencing files and the raw data associated with each figure, have been deposited in the Zenodo database and are accessible to the public through the accession code: 8388902 (https://zenodo.org/records/8388902). Information about the PDB Entry 1C0P (DAOx) are found here: https://www.wwpdb.org/pdb?id=pdb_00001c0p. Additionally, the heatmaps and their associated raw data have been made available in color-blind accessible and interactive formats at the following location: https://nash-lab.github.io/DAOx-DMS/heatmaps/hm_expression_activity.html, https://nash-lab.github.io/DAOx-DMS/heatmaps/hm_normalized_activity.html.

## Code availability

All the custom computer codes and programs required to generate the data of this work are publicly available in the Zenodo repository and accessible through the 10.5281/zenodo.8388902.
