# Supplementary material for: Understanding activity-stability tradeoffs in biocatalysts by enzyme proximity sequencing
Source: Nat Commun. 2024 Feb 28;15:1807. doi: 10.1038/s41467-024-45630-3 (PMC10902396; doi:10.1038/s41467-024-45630-3)
Supplement: Supplementary file 1 — Supplementary Information [file 41467_2024_45630_MOESM1_ESM.pdf]

SUPPLEMENTARY INFORMATION

Understanding Activity-Stability Tradeoffs in Biocatalysts by Enzyme Proximity Sequencing

Rosario Vanella[1, 2, *], Christoph Küng[1, 2], Alexandre A. Schoepfer [1, 3, 4], Vanni Doffini[1, 2], Jin Ren[1, 2], Michael A. Nash[1, 2, 5, 6, *]

[1] Institute of Physical Chemistry, Department of Chemistry, University of Basel, 4058 Basel, Switzerland
[2] Department of Biosystems Science and Engineering, ETH Zurich, 4058 Basel, Switzerland
[3] Institute of Chemical Sciences and Engineering, École Polytechnique Fédérale de Lausanne (EPFL), 1015 Lausanne, Switzerland
[4] National Center for Competence in Research (NCCR), Catalysis, École Polytechnique Fédérale de Lausanne (EPFL), 1015 Lausanne, Switzerland
[5] National Center for Competence in Research (NCCR), Molecular Systems Engineering, 4058 Basel, Switzerland
[6] Swiss Nanoscience Institute, 4056 Basel, Switzerland

* Correspondence to: rosario.vanella@unibas.ch; michael.nash@unibas.ch

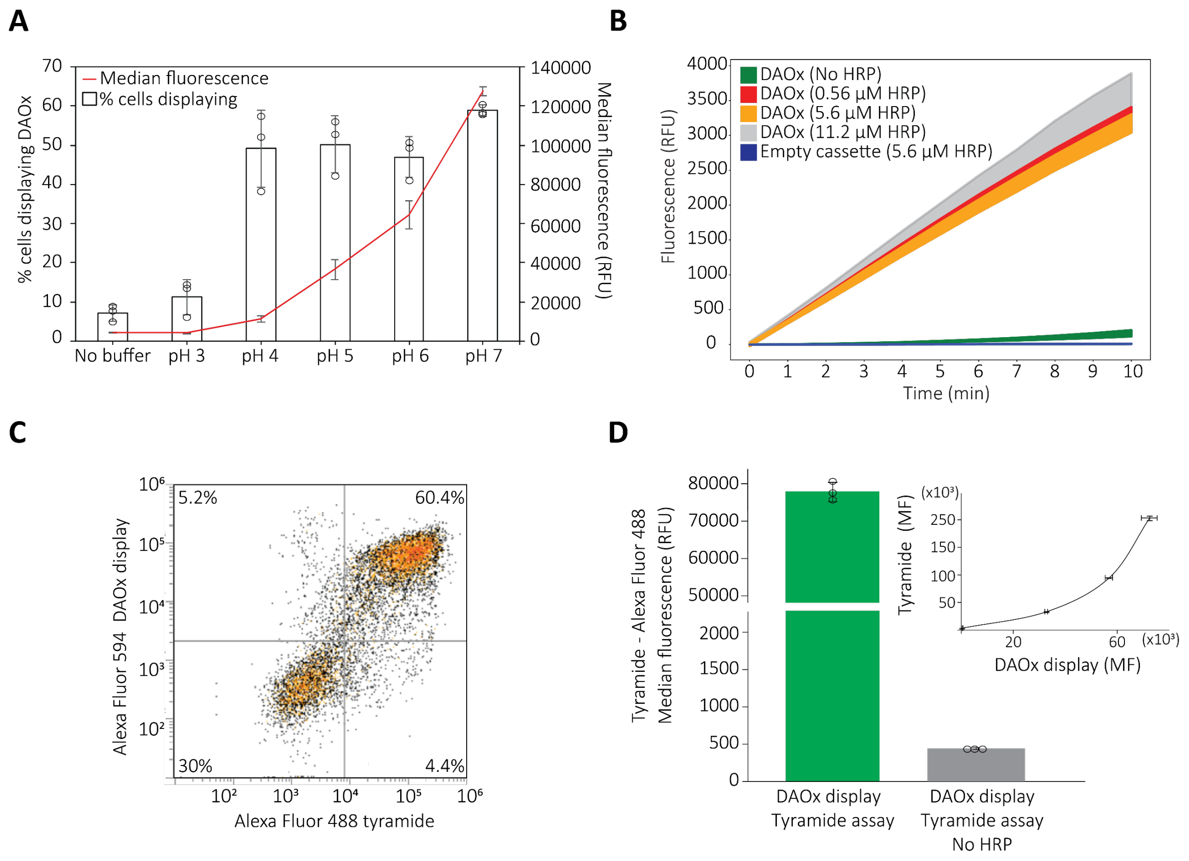

Supplementary Figure 1. Yeast surface display of D-amino acid oxidase from *Rhodotorula gracilis*.

A) DAOx surface display levels after induction of protein expression at 20°C for 48h in growth media at different pHs. Percentage (%) of yeast population displaying DAOx wild type and median fluorescence relative fluorescent units (RFU) of the displaying population are reported (*n*=3 experimental replicates). B) Population based DAOx activity assay. Half a million cells from a DAOx displaying population were used for an Amplex Red activity assay to detect the activity of the displayed enzyme. A reaction using cells not displaying DAOx (empty cassette) and a reaction using DAOx displaying cells but not HRP in the mixture were used as negative controls. The reaction was tested at increasing concentration of HRP in order to verify that HRP concentration was not limiting the rate of the reaction. C) Single cell tyramide activity assay. A population of cells positive for the display of the DAOx wild type enzyme was assayed for the activity of the enzyme through a single cells tyramide assay. The same assay was then used for the screening of the DAOx variant library. D) Tyramide-AlexaFluor 488 fluorescence of a cell population displaying DAOx incubated with the complete tyramide assay reaction mixture and with the reaction mixture lacking HRP (No HRP). The inset shows how the median fluorescence (MF) linked to the surface display of DAOx correlates with the fluorescence gained by the cells assayed through tyramide assay (*n*=3 experimental replicates).

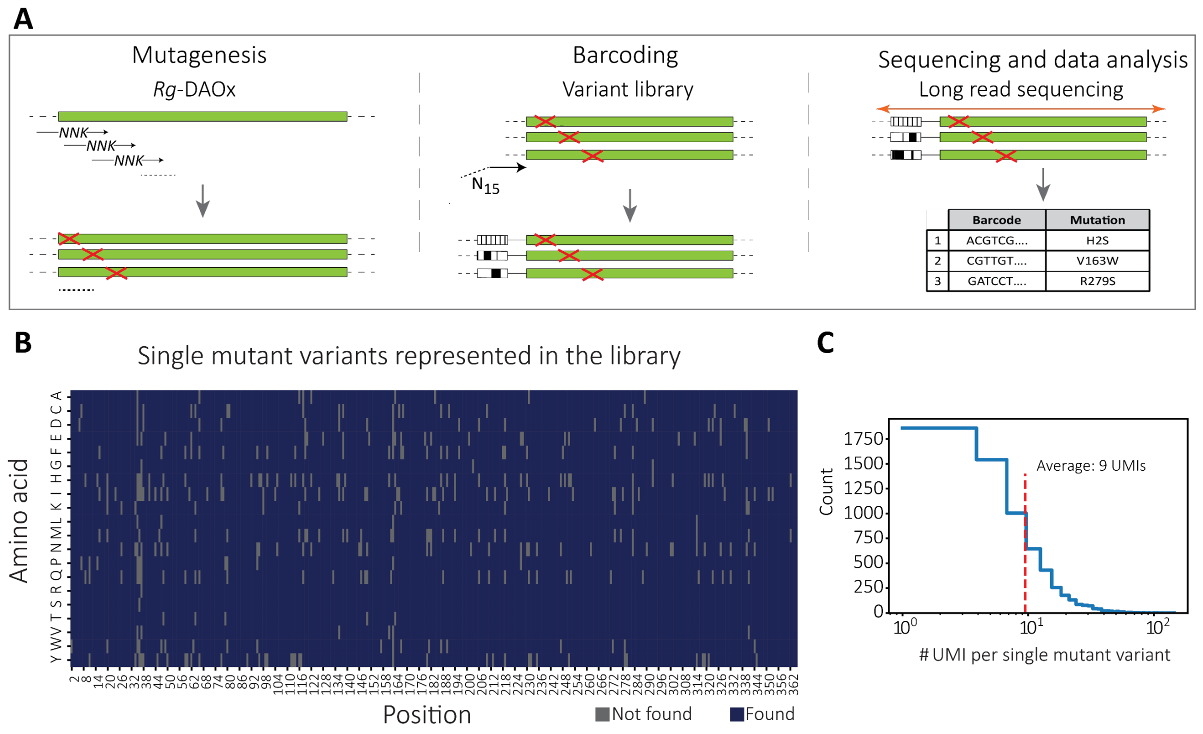

Supplementary Figure 2. Mutant library construction and sequencing.

(A) A variant library of the enzyme DAOx was constructed by means of one pot site saturation mutagenesis[1]. Variants represented in the library were linked to unique molecular identifiers (UMI) by PCR. PacBio long read sequencing was used to reveal the pairs variant-barcode and the information stored in a look up table. (B) 6,530 of 6,916 possible single amino acid missense mutants are represented in the library (94.4% of total). (C) Number of UMIs associated with single mutant missense variants represented in our library.

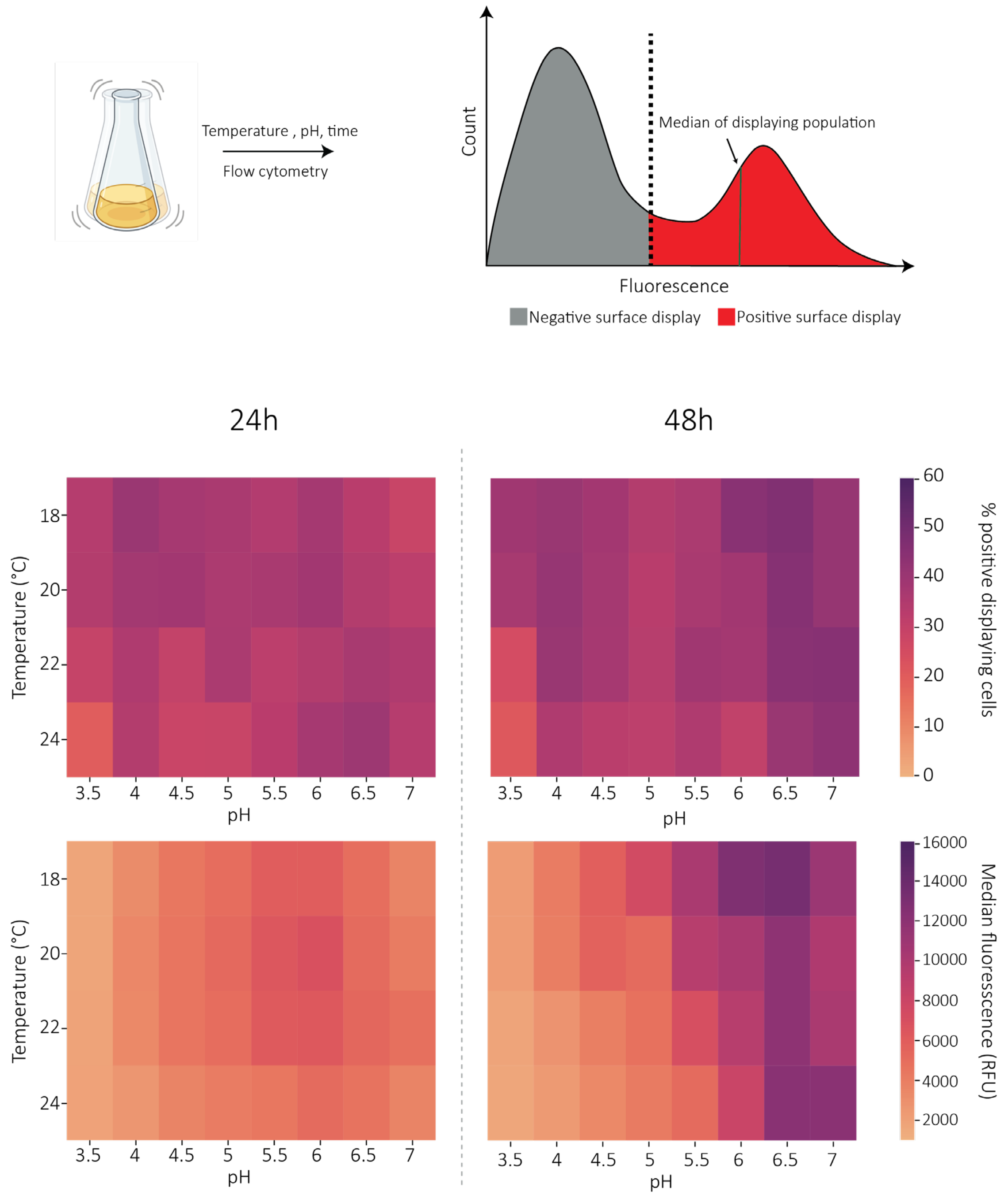

Supplementary Figure 3. Yeast surface display optimization of DAOx mutant library.
The expression and display level of the variant enzymes were tested at 24 and 48h and at increasing temperatures (from 18 to 24°C) and pHs (3.5 to 7) in three independent replicates. The display of DAOx on the surface of yeast cells was detected through fluorescent antibody staining by targeting the C-terminal histidine tag of the Aga2-DAOx fusion construct. The percentage and median fluorescence of the positive displaying population were measured from each experiment and are represented as heatmaps. The peak condition for both the evaluation criteria appears at 48h of protein induction time, pH range 5 to 7 and temperature range between 18 and 20 °C.

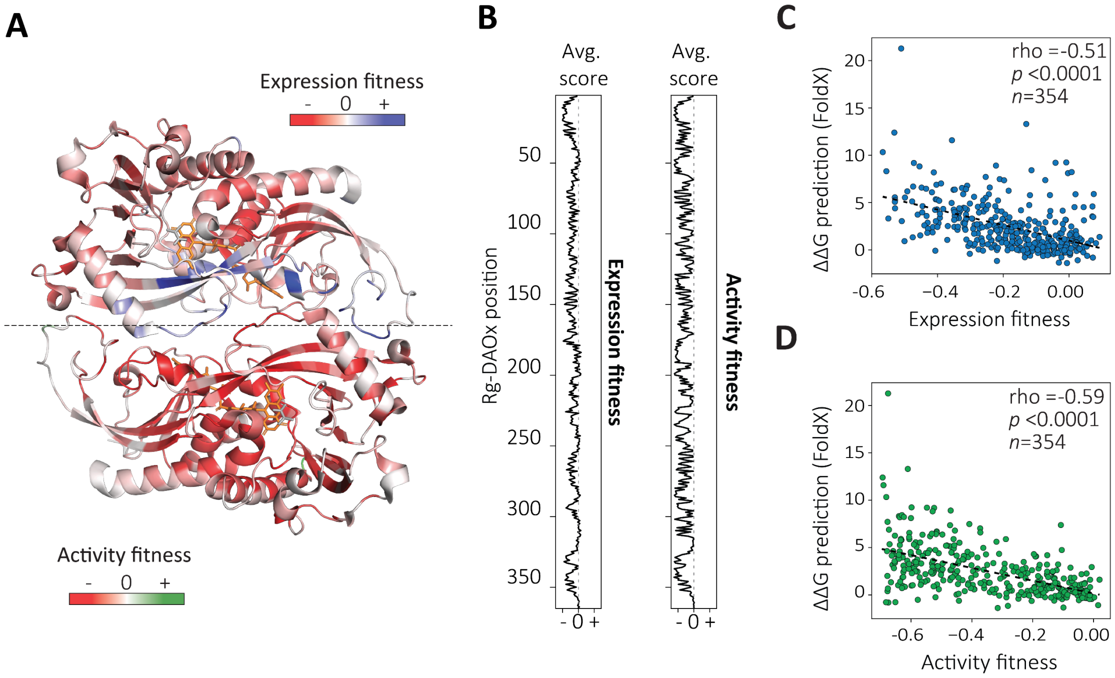

Supplementary Figure 4. Mean fitness expression and activity scores and their correlation with predicted ΔΔG.

A) Mean expression (top) and activity (bottom) fitness scores per position of the DAOx mapped on the 3D structure of the enzyme (PDB: 1C0P). B) Average expression (left) and activity (right) fitness scores along the sequence of DAOx. C) Spearman's rank correlation (two tailed) between predicted ΔΔG values and experimental expression scores per position of the protein. D) Spearman's rank correlation (two tailed) between predicted ΔΔG values and experimental activity scores per position of the protein.

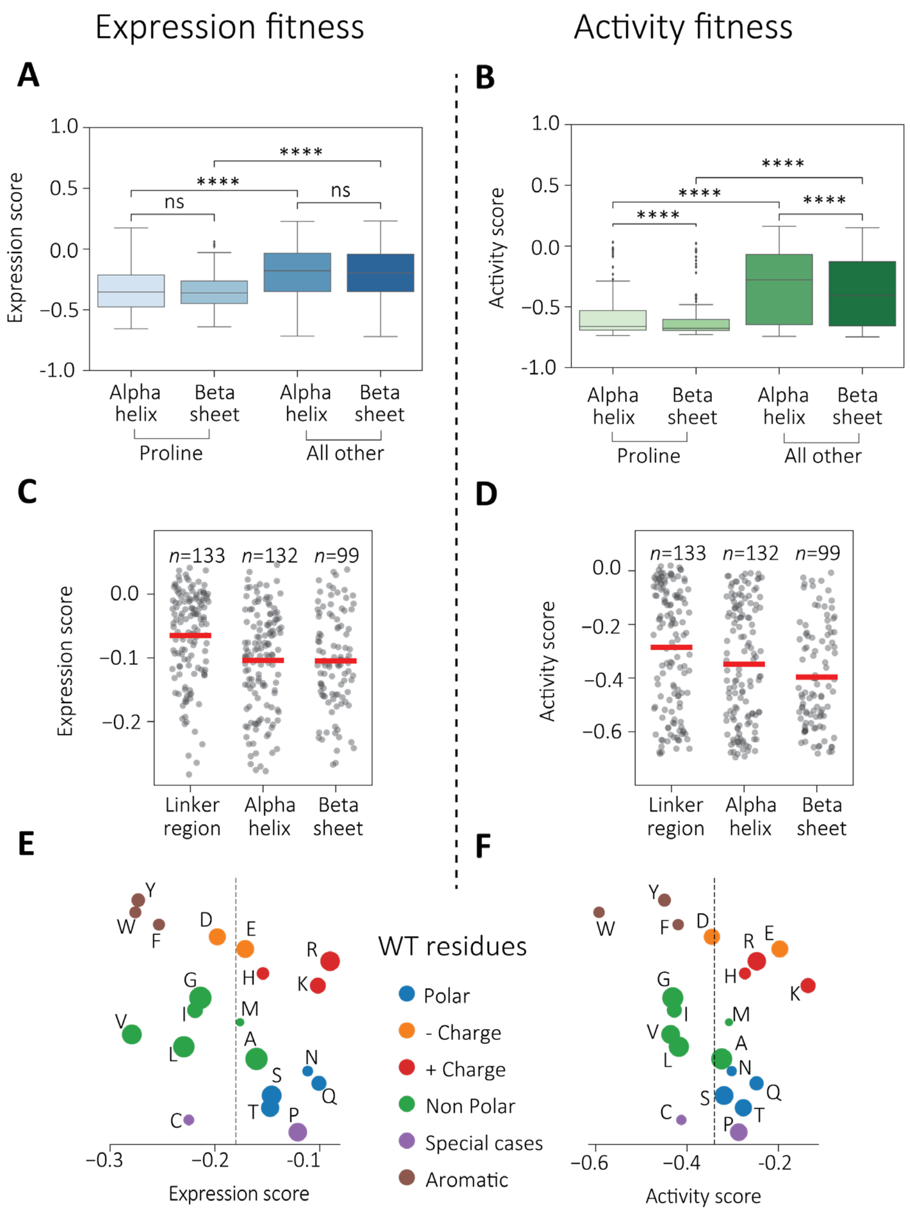

Supplementary Figure 5. Analysis of mutational effects through biochemical properties of amino acids and structural elements of DAOx enzyme.

A) Impact of proline insertion on the expression of DAOx (Mann-Whitney U test, two-sided; ns proline: p=0.9217, ns All other: p= 0.40155, ****: p< 0.0001) B) Impact of proline insertion on the enzymatic activity of DAOx (Mann-Whitney U test, two-sided; ****: p-value< 0.0001). Proline in alpha helix datapoints n= 123, proline in beta sheets datapoints n= 91, other amino acids in alpha helix datapoints n= 2,192, other amino acids in beta sheets datapoints n= 1,669. C) Effect of single amino acid mutations on the expression of DAOx in relation to its secondary structure elements. D) Effect of single amino acid mutations on the enzymatic activity of DAOx in relation to its secondary structure elements. E) Impact of the substitution of wild type amino acids on the expression of DAOx. F) Impact of the substitution of wild type amino acids on the activity of DAOx. Size of the dots represents a relative measure of the amino acid abundance in the wild type sequence of DAOx. The reference fitness score (dashed line) was established as the average fitness values derived from grouping the dataset by the nature of substituted wild-type amino acids, resulting in -0.182 for expression and -0.342 for activity.

A

Property	Expression score dataset Pearson *r* , p-value (*p*)		Activity score dataset Pearson *r* , p-value (*p*)	
WT Bulkiness	**-0.10**	*p* <0.0001	**-0.07**	*p* <0.0001
Mut Bulkiness	**-0.03**	*p* =0.0387	**-0.01**	*p* =0.4261
Δ Bulkiness	**-0.06**	*p* <0.0001	**-0.05**	*p* =0.0003
WT Polarity	**0.19**	*p* <0.0001	**0.25**	*p* <0.0001
Mut Polarity	**-0.02**	*p* =0.1125	**-0.08**	*p* <0.0001
Δ Polarity	**0.14**	*p* <0.0001	**0.22**	*p* <0.0001
WT Hydrophob.	**-0.22**	*p* <0.0001	**-0.26**	*p* <0.0001
Mut Hydrophob.	**0.06**	*p* <0.0001	**0.09**	*p* <0.0001
Δ Hydrophob.	**-0.20**	*p* <0.0001	**-0.25**	*p* <0.0001

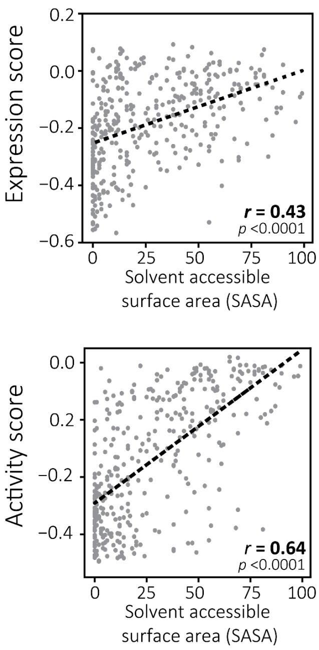

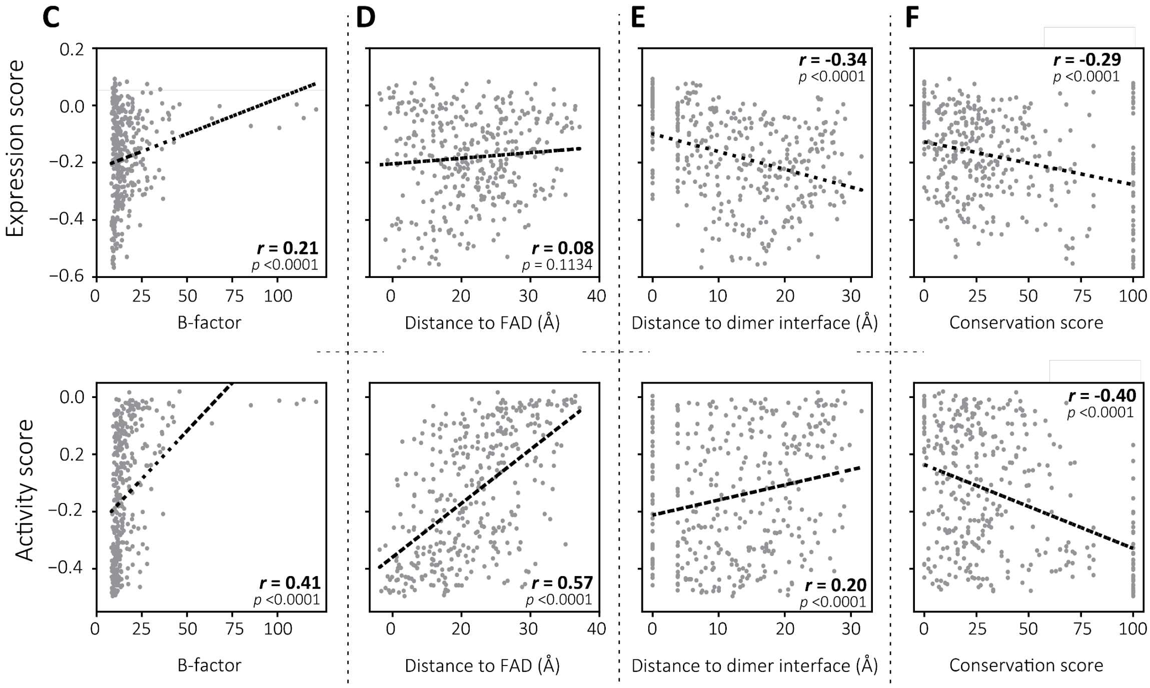

Supplementary Figure 6. Linear correlation analysis of the expression and activity DAOx datasets with biochemical, structural and functional properties of the enzyme.

A) Pearson correlation coefficient (*r*) of activity and expression datasets (*n*=6,399) with chemical physical properties of wild type and mutant amino acids. B) Linear regression of the expression (top) and activity (bottom) datasets with solvent accessible surface area (SASA) score calculated per position of the protein (*n*=360). C) Linear regression of the expression (top) and activity (bottom) fitness scores with temperature factor (B-factor) extracted from the crystal structure data of DAOx (*n*=360). D) Linear regression of the expression (top) and activity (bottom) scores with the distance of the mutation site from the FAD cofactor (*n*=360). E) Linear regression of the expression (top) and activity (bottom) fitness scores with the distance of the mutation site to the dimer interface. All the structural features were extracted from the 3D structure of DAOx (*n*=360) (PDB:

1C0P). F) Linear regression of the experimental expression and activity datasets with natural evolution conservation score calculated by aligning the wild type sequence of DAOx with 5 evolutionary related DAOx protein sequences (*n*=360) (see Supplementary Fig. 7). B-F) The Pearson correlation coefficient was calculated to assess the strength and direction of the linear association. A two-tailed test was employed to determine the significance of the correlation.

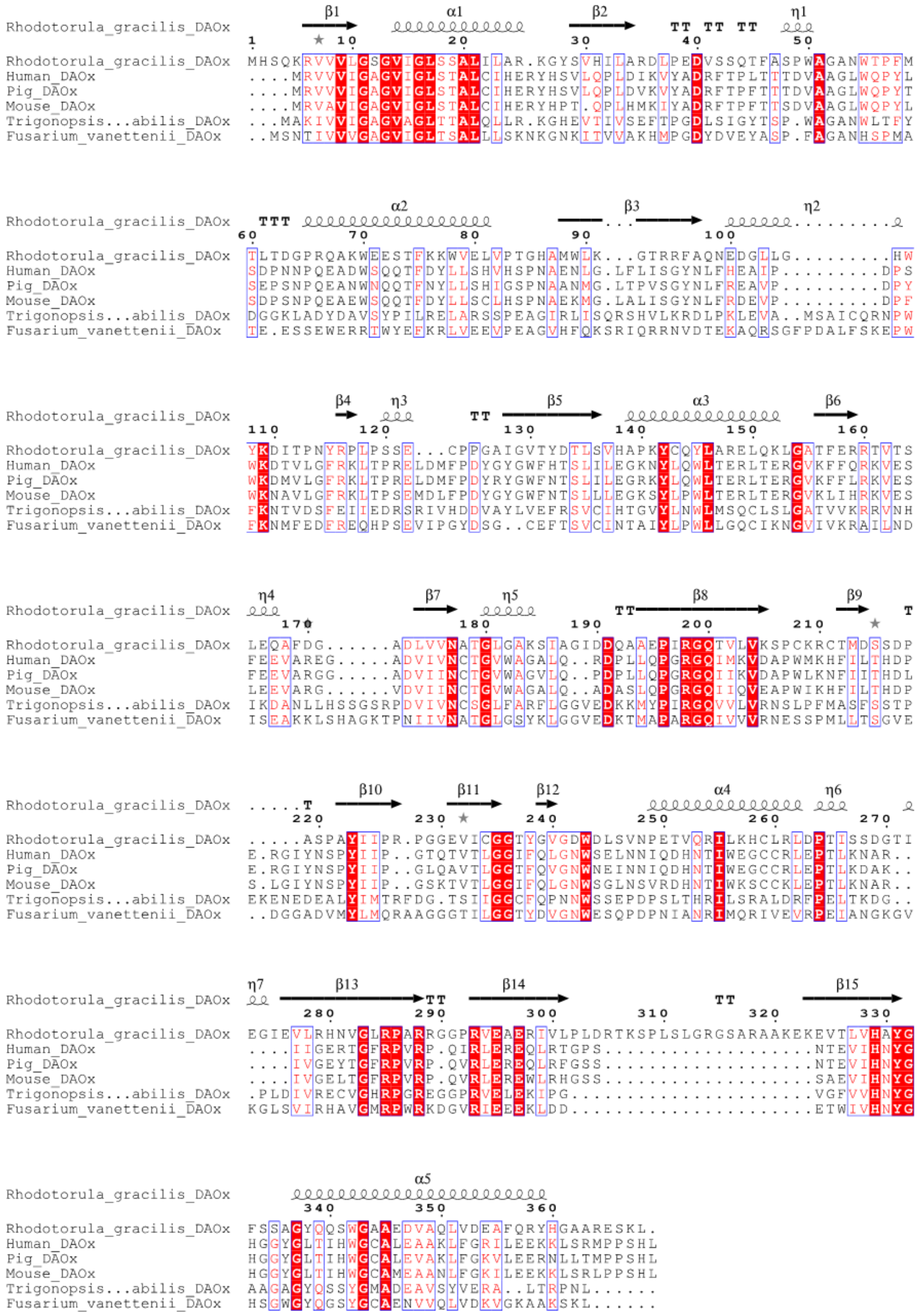

Supplementary Figure 7. DAOx sequences alignment.

Alignment of the protein sequence of *Rhodotorula gracilis* DAOx studied in this work, to evolutionary related D-amino acid oxidase sequences from human, pig, mouse, *Trigonopsis variabilis* and *Fusarium vanettenii* [2]. ClustalX software was used to compute the alignment and extract a conservation score per position of the DAOx protein from *Rhodotorula gracilis.*

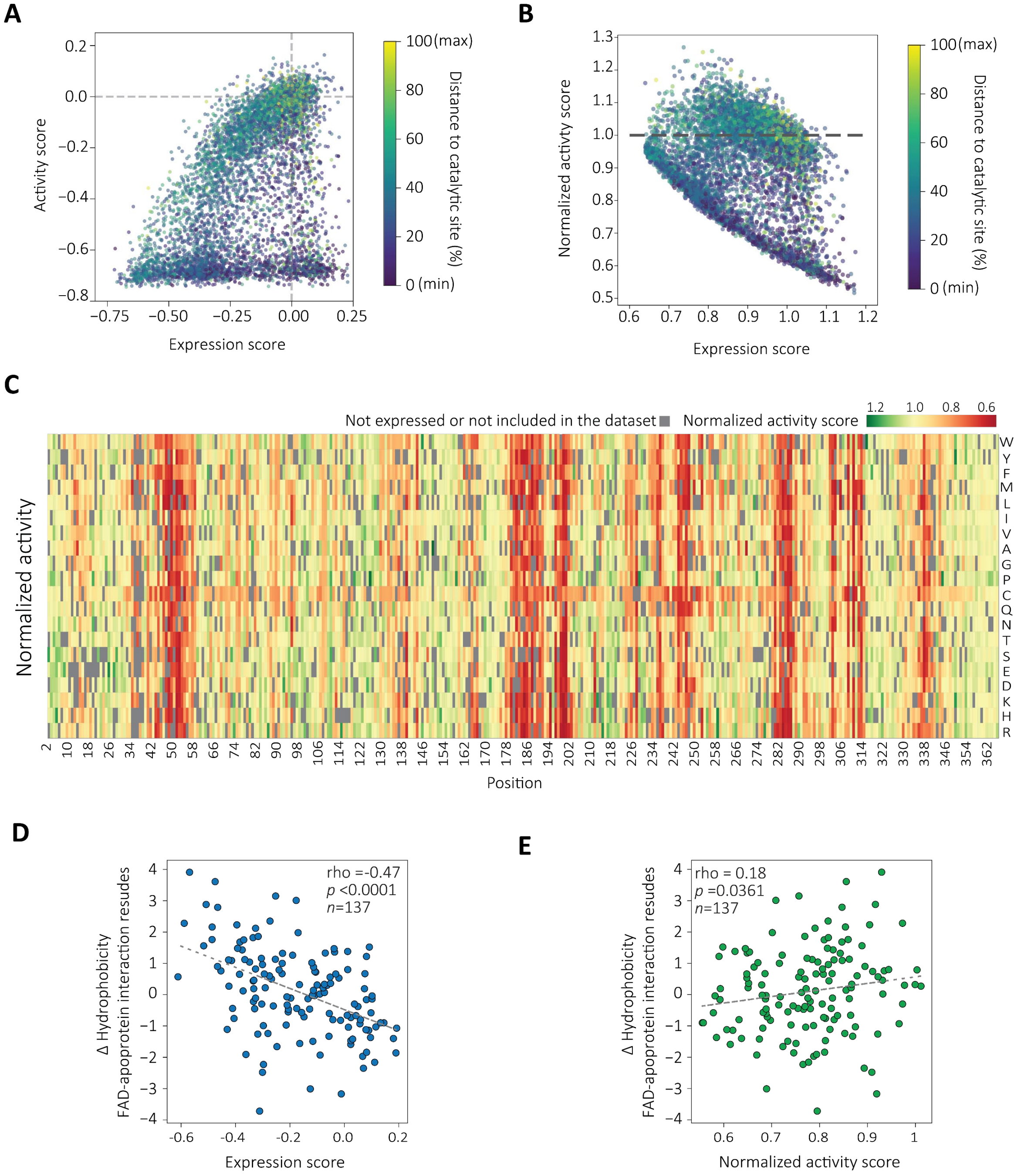

Supplementary Figure 8. Deconvoluting activity from expression level.

A) Scatter plot displaying the relationship between expression and activity fitness scores for 6,325 single mutant variants. B) Activity fitness scores were normalized over expression fitness scores (normalized activity) and visualized in relation to the expression score. Variants with normalized activity values greater than 1 (dashed line) represent single mutant variants where activity was improved independently of the expression level. C) Fitness heatmap of normalized activity scores along the sequence of DAOx. Gray boxes indicate mutant variants excluded from normalization due to their low expression scores (expression score <-0.65) and missing variants in the initial library. Interactive and color-blind accessible version of the heatmap can be found here:https://nash-lab.github.io/DAOx-DMS/heatmaps/hm_normalized_activity.html. D) Spearman's rank correlation (two-sided test) between predicted changes in hydrophobicity at the core of DAOx and expression fitness scores. E) Spearman's rank correlation (two-sided test) between predicted changes in hydrophobicity at

the core of DAOx and normalized activity scores. Delta hydrophobicity values were calculated by subtracting the hydrophobicity score of the mutant residue from that of the wild-type residue for all variants with mutations at positions interacting with the FAD cofactor[3]. Conversely, positive delta hydrophobicity indicates a decrease in hydrophobicity whereas negative delta hydrophobicity indicates an increase in hydrophobicity.

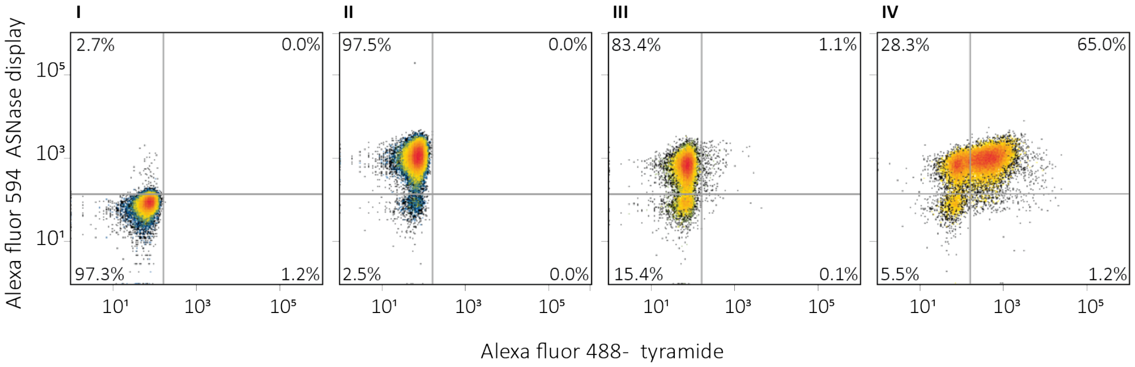

Supplementary Figure 9. ***Escherichia coli*** **surface display and tyramide assay of Asparaginase 2 from *Erwinia chrysanthemi.***

(I) *Escherichia coli* (*E. coli* BL21 DE3) cells induced for the surface expression of *Erwinia chrysanthemi* Asparaginase 2 (ASNase) analyzed through flow cytometry. (II) *E.coli* surface display of ASNase detected through antibody staining and measured through flow cytometry. (III) Single cell tyramide activity assay negative control. A population of cells positive for the display of the ASNase was incubated with tyramide assay reaction mixture (1mM Asparagine, 10 µM *E. coli* Aspartate oxidase, 1/200 dilution of Alexa fluor 488 Tyramide, 50 µM FAD, 1.5% w/v alginate) lacking HRP for 45 minutes at room temperature. Cells were then analyzed through flow cytometry. (IV) Single cell tyramide activity assay. A population of cells displaying ASNase enzyme was assayed for the activity of the enzyme through a single cell tyramide assay. Cells were mixed with 1mM Asparagine, 10 µM *E. coli* Aspartate oxidase, 1 µM HRP, 1/200 dilution of Alexa fluor 488 Tyramide, 50 µM FAD, 1.5% w/v alginate and incubated at room temperature for 45 minutes before being analyzed through flow cytometry. Each experiment was performed in triplicate. We show one of the replicates.

Supplementary Table 1. Look-up table composition.

Nucleotide and amino acid DAOx variants (codons 2-365) registered in the mutant library and associated with unique molecular identifiers (UMI).

	# Variants	# UMI (tot: 173,002)
0 nucleotide mutations (WT)	-	54,158
1 nucleotide mutations	6,530 missense<br>336 nonsense<br>308 synonymous	59,743<br>2,689<br>4,692
2 nucleotides mutations	31,943 missense<br>2,814 nonsense<br>171 synonymous	33,812<br>2,983<br>178
3-5 nucleotides mutations	-	14,747

Supplementary Table 2. Number of sorted cells and Illumina reads.
Information about the number of cells sorted per bin in each of the expression level and activity level screening experiments. Number of Illumina reads per sample and median fluorescence values per sorting gate are also provided.

Experiment	Bin	Median fluorescence	# sorted cells	# Illumina reads
Expression level Replicate 1	# 1	146	6,243,697	42,024,431
	# 2	3,424	1,926,870	39,848,257
	# 3	19,202	1,757,354	41,267,548
	# 4	48,439	1,704,305	41,474,350
Expression level Replicate 2	# 1	145	6,662,430	41,086,125
	# 2	1,938	2,033,831	32,102,375
	# 3	14,289	1,739,456	40,933,088
	# 4	40,094	1,554,250	42,280,563
Activity level Replicate 1	# 1	825	5,526,601	41,466,216
	# 2	4,873	814,188	38,937,993
	# 3	15,295	1,001,782	36,382,700
	# 4	40,787	908,535	38,617,574
Activity level Replicate 2	# 1	812	6,454,983	38,514,974
	# 2	4,897	837,272	41,667,242
	# 3	14,973	1,027,386	42,046,908
	# 4	42,012	933,111	40,046,573

Supplementary Table 3. DNA primers used in this work.
Sequence of NNK primers used to generate the mutant RgDAOx library are provided at: https://zenodo.org/record/8388902

Primer name	5'-3' sequence
F1	GCGCGGCCTTTTGCCCTGCAGGCCNNNNNNNNNNNNNNNAGGGAACAAAAGCTGGCTAGTACGG
R1	CGATTTTGTTACATCTACACTGTTGTTATCAGATCAGCGGGTTTAAAC
F3	TCGTCGGCAGCGTCAGATGTGTATAAGAGACAGGGCCTTTTGCCCTGCAGGC
F4	TCGTCGGCAGCGTCAGATGTGTATAAGAGACAGTGGCCTTTTGCCCTGCAGGC
F5	TCGTCGGCAGCGTCAGATGTGTATAAGAGACAGATGGCCTTTTGCCCTGCAGGC
F6	TCGTCGGCAGCGTCAGATGTGTATAAGAGACAGCATGGCCTTTTGCCCTGCAGGC
R3	GTCTCGTGGGCTCGGAGATGTGTATAAGAGACAGGGAGGAGAGTCTTCCTTCGGAGGG
R4	GTCTCGTGGGCTCGGAGATGTGTATAAGAGACAGTGGAGGAGAGTCTTCCTTCGGAGGG
R5	GTCTCGTGGGCTCGGAGATGTGTATAAGAGACAGATGGAGGAGAGTCTTCCTTCGGAGGG
R6	GTCTCGTGGGCTCGGAGATGTGTATAAGAGACAGGATGGAGGAGAGTCTTCCTTCGGAGGG

Supplementary Table 4. Effect of mutant amino acid on expression and activity scores.
AR= aromatic; CN= charge negative; CP= charge positive; HP= hydrophobic; PU= polar uncharged; SC= special case

Mutant amino acid	Amino acid class	Expression fitness	Expression fitness (% change from average)	Activity fitness	Activity fitness (% change from average)	Count
Y	AR	-0.200	-11.754	-0.374	-11.304	310
F	AR	-0.197	-9.711	-0.354	-5.393	314
W	AR	-0.232	-29.113	-0.393	-16.806	332
D	CN	-0.180	-0.633	-0.391	-16.353	317
E	CN	-0.159	11.321	-0.373	-10.905	308
R	CP	-0.241	-34.273	-0.407	-20.999	337
H	CP	-0.180	-0.273	-0.329	2.016	300
K	CP	-0.218	-21.621	-0.391	-16.460	328
V	HP	-0.143	20.226	-0.280	16.788	328
M	HP	-0.133	25.550	-0.264	21.533	318
A	HP	-0.148	17.482	-0.273	18.700	317
G	HP	-0.190	-5.725	-0.345	-2.689	321
I	HP	-0.149	16.780	-0.269	19.823	298
L	HP	-0.172	4.024	-0.290	13.631	325
N	PU	-0.164	8.523	-0.318	5.399	313
Q	PU	-0.164	8.757	-0.322	4.305	306
S	PU	-0.148	17.291	-0.275	18.238	335
T	PU	-0.139	22.753	-0.283	15.708	333
P	SC	-0.281	-56.884	-0.527	-56.839	328
C	SC	-0.148	17.284	-0.263	21.605	331
	AR	-0.210	-16.859	-0.374	-11.168	956
	CN	-0.170	5.344	-0.382	-13.629	625
	CP	-0.213	-18.722	-0.376	-11.814	965
	HP	-0.156	13.056	-0.287	14.631	1907
	PU	-0.154	14.331	-0.299	10.912	1287
	SC	-0.215	-19.800	-0.395	-17.617	659

Supplementary Note 1 - Cloning and yeast surface display of D-amino acid oxidase (DAOx).

To develop our workflow on DAOx, we codon optimized the cDNA sequence for *Saccharomyces cerevisiae,* cloned it downstream of the Aga2 display anchor in a pYD1 derived plasmid, and transformed into *Saccharomyces cerevisiae* EBY100. The fusion construct further contained a 6x-Histidine tag at its C-terminal end for antibody labeling. Yeast cells were cultured in galactose rich buffered media at 20°C for 48h. We found pH 7 to be optimal for Aga2-DAOx surface display, with more than 60% of the yeast population expressing WT DAOx under these conditions (Supplementary Fig. 1A). To confirm correct folding and assembly of dimeric DAOx on the yeast surface, we tested the activity of displayed WT DAOx on D-alanine using a coupled HRP/Amplex Red assay. We detected DAOx activity for 500,000 cells displaying WT DAOx, with no fluorescence detected for yeasts lacking the displayed enzyme (Supplementary Fig. 1B). To saturate DAOx and detect the maximum reaction rate, we used D-alanine at 35 mM, which was >40-fold higher than the reported K_m of 0.8 mM[4], and ~5-fold higher than the K_m we measured both for yeast displayed (7.316 ± 0.2816 mM) and soluble WT DAOx (6.965 ± 0.4003 mM). We varied HRP concentration in the assay and verified that, at an HRP concentration of 5.6 µM, the oxidation of D-alanine by DAOx was the rate limiting step of the reaction (Supplementary Fig. 1B). Next, we developed the single cell tyramide/peroxidase proximity labeling assay to measure DAOx activity in a format that would be compatible with pooled screening and DMS[5–7]. We mixed yeast cells displaying DAOx with 130 mM D-alanine, a 1/200 dilution of fluorescent tyramide-488, and 56.5 µM HRP. DAOx activity on D-alanine generated one equivalent of H_2O_2, which served as a downstream substrate for HRP-mediated proximity labeling of the yeast surface by tyramide-488. We observed a strong increase in green fluorescent signal for DAOx-expressing cells, while non-expressing cells showed no such increase (Supplementary Fig. 1C). The specificity of the reaction was confirmed by incubating WT DAOx yeast in a reaction mixture lacking HRP, and detecting a more than 200-fold lower median fluorescence (Supplementary Fig. 1D). We found that tyramide fluorescence of cell population was correlated with red fluorescence (expression stain) in the same population, demonstrating a quantitative relationship between DAOx display levels and tyramide labeling intensity (Supplementary Fig. 1D inset).

Supplementary Note 2 - Library construction and barcoding.

To construct a library for DMS analysis, we used one-pot site saturation nicking mutagenesis over the entire coding region of DAOx (codons 2 to 365)[1]. To overcome the read length limitation of Illumina sequencing, we barcoded the variant library with 15 nucleotide unique molecular identifiers (UMIs) such that each variant was linked to one or more UMIs. The link between UMIs and variants was established through long read Pacbio sequencing (Supplementary Fig. 2A). We estimated the total size of our library to be approximately 200,000 variants and stored the information for each UMI and the corresponding DAOx variant in a look-up table. The final UMI barcoded mutant library included nearly all possible DAOx single missense mutations (6,530 in total, 94.4% of theoretical 6,916) (Supplementary Fig. 2B). Each mutant was linked to at least one UMI, with an average of 9 unique UMIs linked to each individual amino acid missense variant (Supplementary Fig. 2C). In addition, our look-up table contained over 50,000 unique mutants carrying between 2 and 5 nucleotide mutations (Supplementary Table 1). In the remainder of this work, we excluded all higher order mutants from the analysis and report on analysis only of single mutants. We tested the surface display of the variant library (Supplementary Fig. 3), and based on the best expression conditions for both the library and WT DAOx, we selected 48h, 20°C and pH 7 as the final expression protocol for the screening stage.

Supplementary Note 3 - Impact of mutant residue identity on the DAOx expression and enzymatic activity

Using data from the 6,399 single missense variants of DAOx for which both expression and activity scores were available, we grouped variants based on identity of the mutant residue and calculated their average effect on expression and activity fitness (Fig. 3A, B, Supplementary Table 4). We plotted the percent fitness change relative to the average of all missense variants (Avg. Exp=-0.180, Avg. Act=-0.336). This analysis showed that proline (P) insertion had the most deleterious effect on both expression and activity, with average expression and activity scores ~57% (Exp=-0.281, n=328; Act=-0.527, n=328) lower than the average fitness of all variants in the respective screen. Cysteine (C) had a much less deleterious effect (~ +19% of average) on both properties (Exp=-0.148, Act= -0.263, n=331). We further found that neutral polar residues had a less deleterious effect on expression and activity fitness than the average mutation (Exp=-0.154, ~ +14% Exp; Act=-0.299, ~ +10%; n=1287). Among non-polar residues, glycine (G) was the only substitution with lower than average fitness in both screens (Exp=-0.190, -6%; Act= -0.345, -3%; n=321). All other non-polar residues (leucine(L), valine (V), methionine (M), alanine (A) and isoleucine (I)) had an overall positive influence on expression and activity, with an average expression score ~17% and activity score of ~18% higher than the average of all missense mutations (Exp=-0.149; Act=-0.275). Insertion of charged amino acids except for histidine (H) had negative effects on catalytic activity with a fitness score ~12% lower than the average (Act=-0.379). Among charged residues glutamic acid (E) substitutions resulted in higher than average expression of +11% (Exp=-0.159; n=308) despite the general trend of charged amino acids having a negative impact on expression (Exp=-0.1900, -7%, n=1590). Among hydrophobic residues, bulky tryptophan (W) impaired the most both expression and activity fitness (Exp=-0.232, -29%; Act=-0.393, -17%; 332 variants) (Fig. 3A, B, Supplementary Table 4).

Supplementary Note 4 - Impact of the 3D structure on the expression and activity of DAOx

We next investigated whether the negative effects of proline insertion on DAOx expression and activity might be related to its ability to disrupt α helix and β sheet secondary structures due to the missing amide hydrogen necessary for hydrogen bonding[8,9]. We found that inserting proline into α helix and β sheet regions of the DAOx significantly impaired both the expression and activity fitness compared to the effect of any of the other 19 amino acids when inserted in the same regions (Expression:α helix, Mann–Whitney U=85824.0, $p<0.0001$, β sheet, Mann–Whitney U=44911.0, $p<0.0001$; Activity: α helix, Mann–Whitney U=26334.0, $p<0.0001$, β sheet, Mann–Whitney U=13744.0, $p<0.0001$, Supplementary Fig. 5A,B). We expected regions of the protein possessing different secondary structures to have different tolerance levels for mutation that would be reflected in the expression and activity scores. We evaluated the average expression and activity fitness as a function of secondary structure of the mutated position and observed that linker regions had greater tolerance to mutations in both expression (avg. Exp=-0.129) and activity (avg. Act=-0.286) screens compared to structured regions (α helix, avg. Exp=-0.208, avg. Act=-0.348; β sheet, avg. Exp=-0.210, avg. Act=-0.396). β sheet regions had consistently the lowest tolerance to mutations (Supplementary Fig. 5C,D).

Supplementary Note 5 - Impact of wild type amino acid substitution on the DAOx expression and enzymatic activity

We analyzed expression and activity fitness as a function of the substituted wild type amino acids (Supplementary Fig. 5E,F) and found that mutating low abundance hydrophobic core aromatic residues such as tryptophan (W) and tyrosine (Y) together with valine (V), one of the most abundant residues in DAOx (*n*=27), had the highest impact on the expression of the enzyme (avg. Exp V,Y,W=-0.276; avg. Exp all=-0.182) (Supplementary Fig. 5E). Catalytic activity was strongly negatively affected by substitution of tryptophan (W) (avg. Act W=-0.593; avg. Act all= -0.342) (Supplementary Fig. 5F). Mutation of polar or charged amino acids was comparatively less deleterious and was associated with on average higher scores (avg. Exp=-0.136, +25%; avg. Act,-0.260, +23%) than the other classes of amino acids (avg. Exp= -0.220; avg. Act=-0.410, Supplementary Fig. 5E,F).

Supplementary References

1. Wrenbeck, E. E. *et al.* Plasmid-based one-pot saturation mutagenesis. *Nat. Methods* **13**, 928–930 (2016).
2. Robert, X. & Gouet, P. Deciphering key features in protein structures with the new ENDscript server. *Nucleic Acids Res.* **42**, W320-4 (2014).
3. Pollegioni, L. *et al.* Yeast D-amino acid oxidase: structural basis of its catalytic properties. *J. Mol. Biol.* **324**, 535–546 (2002).
4. Sacchi, S. *et al.* Engineering the substrate specificity of D-amino-acid oxidase. *J. Biol. Chem.* **277**, 27510–27516 (2002).
5. Becker, S. *et al.* Single-cell high-throughput screening to identify enantioselective hydrolytic enzymes. *Angew. Chem. Int. Ed Engl.* **47**, 5085–5088 (2008).
6. Lipovsek, D. *et al.* Selection of horseradish peroxidase variants with enhanced enantioselectivity by yeast surface display. *Chem. Biol.* **14**, 1176–1185 (2007).
7. Ostafe, R., Prodanovic, R., Nazor, J. & Fischer, R. Ultra-high-throughput screening method for the directed evolution of glucose oxidase. *Chem. Biol.* **21**, 414–421 (2014).
8. Imai, K. & Mitaku, S. Mechanisms of secondary structure breakers in soluble proteins. *Biophysics* **1**, 55–65 (2005).
9. Gray, V. E., Hause, R. J. & Fowler, D. M. Analysis of Large-Scale Mutagenesis Data To Assess the Impact of Single Amino Acid Substitutions. *Genetics* **207**, 53–61 (2017).
