# Supplementary material for: Understanding activity-stability tradeoffs in biocatalysts by enzyme proximity sequencing
Source: Nat Commun. 2024 Feb 28;15:1807. doi: 10.1038/s41467-024-45630-3 (PMC10902396; doi:10.1038/s41467-024-45630-3)
Supplement: Supplementary file 2 — Peer Review File [file 41467_2024_45630_MOESM2_ESM.pdf]

nature portfolio

Peer Review File

Open Access This file is licensed under a Creative Commons Attribution 4.0 International License, which permits use, sharing, adaptation, distribution and reproduction in any medium or format, as long as you give appropriate credit to the original author(s) and the source, provide a link to the Creative Commons license, and indicate if changes were made. In the cases where the authors are anonymous, such as is the case for the reports of anonymous peer reviewers, author attribution should be to 'Anonymous Referee' followed by a clear attribution to the source work. The images or other third party material in this file are included in the article's Creative Commons license, unless indicated otherwise in a credit line to the material. If material is not included in the article's Creative Commons license and your intended use is not permitted by statutory regulation or exceeds the permitted use, you will need to obtain permission directly from the copyright holder. To view a copy of this license, visit http://creativecommons.org/licenses/by/4.0/.

REVIEWER COMMENTS

Reviewer #1 (Remarks to the Author):

The authors present a new and generalizable method to simultaneously map sequence to enzyme expression and catalytic activity. The approach combines yeast surface display of enzymes, tyramide-based proximity labeling, and next generation sequencing. They apply their method to D-amino acid oxidase (DAOx) and perform an in-depth analysis of the resulting data to understand the biophysical basis of enzyme function. The work is interesting and provides a general platform for high-throughput screening of enzymes.

- The biggest missing piece of information from the paper is scatter plot between expression and activity for all single mutants. Something like Fig 5a but for individual mutations rather than averaging over each site. This would help clarify many of my questions below.

- The tyramide measurement is not capturing pure enzyme activity, but "total activity" that is influenced by protein expression. Based on the FACS gates on Fig 2c, it seems the non-displaying population is gated out, but more subtle differences in expression/display level would influence the activity measurement. Do the authors have thoughts on how much the activity fitness is being influenced by expression level? I found it surprising how strongly the activity score correlated with FoldX and the fact that proline mutations were highly deleterious. This may suggest the activity score is still largely influenced by expression. In the most ideal scenario, you would be able to assess enzyme activity independent of expression level. Is there some way the activity score could be normalized (divided by?) the expression score to provide such a quantity?

- I read through the section "Thermodynamic stability shapes both expression and activity landscapes," but didn't see any connection to thermodynamics. The section title may be more accurately described with "Biophysical properties" or "Physicochemical properties."

- Line 347 (and below): the correlation coefficients may be missing a decimal? (Exp r=-26)

- This is a very nice method that can plug into the many established coupled enzyme assays that utilize oxidases. The Asparginase in Fig S8 was great. In the Discussion, I think it would be worthwhile to list a few more diverse classes of enzymes that could (theoretically) be assayed with this method.

Reviewer #2 (Remarks to the Author):

Summary

In this manuscript, Vanella, et al. develop a method called EP-seq to dissect the relationships between enzyme activity and stability in a high-throughput manner, building upon existing strategies for Yeast Surface Display (YSD), Deep Mutational Scanning (DMS), peroxidase-mediated radical labelling, and FACS. The authors create a DMS library of all possible single mutations of the enzyme D-amino Acid Oxidase (DAOx) and use two parallel assays: one to measure expression and another to estimate enzymatic activity. The resulting Expression Fitness and Activity Fitness scores are analyzed, compared, and mapped onto the three-dimensional structure of DAOx to explore their effects on its stability and activity.

I agree that there is a clear and pressing need for quantitative assays that can deconvolute enzyme activity and stability in high throughput. A fundamental understanding of this relationship would be transformative for biotechnology, medicine, and fundamental biology and would be generally interesting and impactful. My primary concern is that this assay cannot achieve the stated goal of this manuscript, which is to provide "biophysical insights into protein sequence, stability, and function" as there are caveats and biochemical oversimplifications in the creation and interpretation of this assay (see Major Points). In its current form and presentation herein, there is not enough evidence that this method alone has the quantitative power to deliver the biophysical insights that the authors seek. Perhaps the authors should consider presenting this method as a way for selection of more active and better expressed DAOx variants through multiple cycles of this assay, rather than as a tool for biochemical and biophysical dissection of activity-stability tradeoffs. The current motivation for the

method requires a true deconvolution of activity and stability and data rooted in biochemical principals. For an engineering-focused motivation, detailed biochemical analyses could be performed on several final variants from the selection, whereas the current motivation of this method requires more controls to rigorously benchmark these assays against true biochemical and biophysical parameters.

The comments below come from my perspective as an enzymologist and I sincerely hope that they are helpful to the authors.

Major Points

1. What evidence do the authors have that the population of DAOx is correctly dimerized on the yeast surface? I am generally concerned about the use of a cytosolic, dimeric protein for these assays. For dimeric enzymes, proper folding is often coupled with dimerization and the folding of one subunit can be cooperative with the other. Since DAOx is trafficking through the yeast secretory pathway as a monomer (something that it hasn't evolved to do) and unfolded and misfolded proteins are degraded prior to display and dimerization on the surface, observed changes in abundance may not reflect true thermodynamic stability [i.e. the Gibbs free energy difference between the native dimerized state to the unfolded state(s)]. The expression measurement may instead represent the "stability" of an aberrant misfolded species or intermediate species not normally observed in high frequency when the protein folds in the cytosol. Since the trafficked protein is likely exposed to the protein degradation machinery as a monomer prior to surface display and dimerization, it is unlikely that the expression differences reported herein represent the thermodynamic stability of the native, dimerized state. Given these considerations, I think a rigorous definition of what the authors mean by stability is needed. Prior YSD studies referenced in this manuscript focused on TEM-1 beta-lactamase and Levoglucosan kinase, both monomeric enzymes (PMID: 28196882). The SARS-CoV-2 receptor binding domain (PMID: 32841599) is also monomeric.
More quantitatively, how much of the displayed DAOx is dimerized? The authors report that they measure a KM for their displayed WT DAOx with D-Ala of 5.01 ± 0.33 mM which is 6-fold higher than the reported KM of 0.8 mM. They do not show the curve this KM value was estimated from, making it challenging to assess the quality of this data. This KM discrepancy is concerning because it could be caused by incorrectly folded WT DAOx or the presence of a competitive inhibitor. Can the authors estimate the percentage of DAOx that is correctly dimerized based on the dimerization Kd and the surface concentration? Is there a way to measure this on the surface? And could the low activities of poorly expressed mutants be due to insufficient concentration to drive dimerization, rather than catalytic impairment?
2. Furthermore, these two studies (PMID: 32841599 & 28196882) are cited in this manuscript to support the claim that YSD abundance of proteins is correlated to thermodynamic stability. This relationship between YSD abundance and thermodynamic stability, while likely holds generally, is also likely to break down in many instances and is not supported by enough in vitro data to quantitively understand the strength and shape of this relationship and where it does not hold. Situations where this relationship may not hold is with dimeric proteins with cooperative folding, as noted in the point above, and with proteins with complicated folding pathways where YSD abundance may be more sensitive to the kinetics of the process than the thermodynamics. What is known about the folding pathway of DAOx and is it likely to be affected by these other features? This comment could also be addressed by redefining the stability as "cellular stability" rather than thermodynamic stability, as the latter has a more rigorous, biochemical definition, or by measuring thermodynamic stability for a number of mutants to benchmark this assay.
3. What sensitivity to differences in activity do the authors expect to get from an assay like this? What is the dynamic range? Over the 45-minute incubation with substrate, how much of the substrate do the authors expect to be consumed? Do they expect to still be in the initial rate regime for all variants? The authors have chosen an enzyme, DAOx, for this study that is exceptionally fast–kcat of 43,250 min-1 (PMID: 1348188). In Figure S1B, half a million cells displaying WT DAOx were used to benchmark the assay (although it is unclear at what concentration) and the reaction already appears to be out of the initial linear range after 10 minutes. The challenge of a pooled assay like this is that faster or better expressed enzymes will consume substrate quickly, starving lower-expressed or less active variants of substrate and artificially lowering their observed activity. This is perhaps why the correlation between the single clone score and the Activity Fitness score (Fig 2G) is unremarkable and is worse than that observed for the Expression Fitness assay. In addition, 35 mM D-Ala is only 7-fold above the observed KM. One could imagine that mutations would increase KM from 5 mM to

something where 35 mM is no longer saturating, adding an additional level of complexity to the interpretation of this data.

4. Related to comment #3, the authors do not normalize their activity fitness assay by abundance. This means they cannot distinguish between variants that are well expressed, but comparatively less active from those that are more active but comparatively less expressed. This convolution is likely why the authors see nearly as good of a correlation between FoldX ΔΔG predictions and their Expression Fitness score as they see for FoldX ΔΔG predictions and their Activity Fitness scores. The authors do not show if there is a correlation between their Expression Fitness score and their Activity Fitness scores, but this would be expected purely from the lack of abundance normalization for their activity measurements. It is critical for the stated goal of this manuscript for the assay to be able to deconvolute activity and expression. Consider normalizing activity scores by expression score to distinguish between activity and stability in analysis.

5. Related to comments #3 and #4, the most novel aspect of this manuscript is the activity assay. The gold-standard for enzyme activity measurement is cellular expression, purification, and measurement of kcat and KM for enzyme variants. This assay should be benchmarked against this gold standard.

6. The authors state: "Only 5 positions (~1%) were found with positive activity fitness and negative expression fitness (Fig. 5A, top left)." This result could be due to the convolution of expression and activity in their Activity Fitness assay and/or due to the pooled format of this activity assay where fast, well-expressed variants compete with lowly-expressed variants for substrate.

7. The authors state: "This shows that mutations at the catalytic site tended to harm activity but improve stability (avg. Exp=-0.068, +12%, avg. Exp all=-0.077, Fig. 3DI), suggesting the enzyme pays a thermodynamic price to remain catalytically active under conditions of functional selection." The authors are describing a well-documented phenomenon stemming from the seminal work of Shoichet and Matthews (PMID: 7831309) and is one of the foundational studies that suggested activity-stability tradeoffs. This should not be presented as a new model and Shoichet and Matthews should be cited.

8. I do not see the noteworthiness of the following statement since it would be predicted from the conservation: "Noteworthy was an N-terminal region (residues 8-32) found to be highly intolerant to amino acid substitutions. This suggests it plays a role in folding stability and could act as an N-terminal intra-molecular chaperone48. The Rossmann fold is highly conserved in this region".

9. It is unclear whether weighting scores by the number of cells is statistically appropriate. I am concerned that in an assay like this, with variations in number of cells and surface expression between different DAOx variants, a weighted mean would skew the interpretation of the data (the authors note this as a positive though, saying "This allowed us to place more emphasis on fitness values that were represented by a higher number of cells in an experimental replicate, resulting in final consensus fitness scores that were calculated with higher confidence"). Can the authors explain in more detail in the methods why this is a sounder statistical metric for this assay rather than a simple mean and doesn't serve to minimize assay error?

10. I am concerned that the conclusions of this paper are too general or already known. For example, the authors state "This indicates that in order for a variant to be catalytically active, it must first be stably expressed and secreted to the cell wall. We therefore observed an expression level-dependency of the catalytic activity." This is a requirement of their assay and central to all of enzyme kinetics (i.e., more enzyme, greater velocity), not a new result or conclusion. The authors state: "We found that WT residues in close proximity to the FAD cofactor in the active site tended to destabilize the enzyme, and could be mutated to enhance folding stability, however this stability was achieved at the cost of catalytic activity." This general concept was described nearly 20 years ago by Shoichet and Matthews (see comment #7). In the context of a new system like DAOx, it would be generally interesting if the authors were able to provide mechanistic and biophysical explanations for why there is an activity-stability tradeoff at these positions.

Minor Points

• Use of word "Fitness": we are unsure if it is appropriate to use the word fitness in this context (i.e., activity fitness score) as the central premise of this paper is to decouple activity from a fitness selection
• Fig 1, 2, 3: consider switching away from red-green colorscale for accessibility
• Fig 2 F/H: what do the vertical dashed lines represent?
• Fig 2 I/K: heatmaps as very difficult to interpret in current formatting, consider allocating more space to them and rotating them horizontally (perhaps J/L could be moved to supplement?)
• Page 7, para 2: who's whose

- Fig 4B: Consider coloring D-ala backbone differently from FAD backbone
- Page 12, para 1: “This demonstrates structure-function tradeoffs at play” should this say stability-function tradeoff?
- Page 13, para 1: how is distance to interface calculated? Can this be 0?
- Page 13, para 1: “quaternary” is unnecessary

General comments for all the reviewers:

We express our gratitude to the reviewers for providing us with valuable comments and insightful suggestions. We have made concerted efforts to address each of the raised comments.

In the revised version of our manuscript, we have taken the following steps to enhance the accuracy and reliability of our study:

1. We repeated the entire experimental process using an improved and carefully curated library of single mutants of the RgDAOx enzyme. This new library still ensures that we cover a comprehensive range of single mutant variants of the target enzyme, resulting in a total size of approximately 200,000 uniquely barcoded variants. This reduced size in comparison to the original library size of over 1 million barcoded variants allows for better control and accuracy of our experimental replicates.
2. We optimized the tyramide assay protocol to improve its sensitivity in detecting the activity of the displayed enzymes at the single-cell level.
3. Additionally, we made minor adjustments to the settings used for cell sorting to ensure that no variants are excluded from our investigation.

These modifications have significantly enhanced the quality of our results. It is important to note that the updated results do not substantially differ from those presented in the original version of our manuscript. This reaffirms the reliability of our dataset, serving as an additional quality check.

We have incorporated all of these changes into the updated version of our manuscript. Figures that required adjustments have been updated accordingly and are included in the revised manuscript. Moreover, part of the final paragraph "Activity enhancing hotspots are globally encoded" was updated according to the new results.

Further details about the new and improved version of our manuscript will be provided also in the following sections, where we address the specific comments raised by the reviewers in a point-by-point manner.

The reviewer's queries are highlighted in black while our corresponding responses are in blue.

Reviewer #1:
The authors present a new and generalizable method to simultaneously map sequence to enzyme expression and catalytic activity. The approach combines yeast surface display of enzymes, tyramide-based proximity labeling, and next generation sequencing. They apply their method to D-amino acid oxidase (DAOx) and perform an in-depth analysis of the resulting data to understand the biophysical basis of enzyme function. The work is interesting and provides a general platform for high-throughput screening of enzymes.

We thank the reviewer for the overall positive evaluation of the work.

The biggest missing piece of information from the paper is the scatter plot between expression and activity for all single mutants. Something like Fig 5a but for individual mutations rather than averaging over each site. This would help clarify many of my questions below.

We have incorporated the scatter plot of the activity score vs. the expression score for each analysed variant in Figure S8A of the manuscript. We also included in the same figure a scatter plot of the activity score normalized to expression vs. expression score (panel B). The raw data that underlies these two plots were deposited on Zenodo and can be accessed with the following DOI:10.5281/zenodo.8388902

The tyramide measurement is not capturing pure enzyme activity, but "total activity" that is influenced by protein expression. Based on the FACS gates on Fig 2c, it seems the non-displaying population is gated out, but more subtle differences in expression/display level would influence the activity measurement. Do the authors have thoughts on how much the activity fitness is being influenced by expression level? I found it surprising how strongly the activity score correlated with FoldX and the fact that proline mutations were highly deleterious. This may suggest the activity score is still largely influenced by expression. In the most ideal scenario, you would be able to assess enzyme activity independent of expression level.

In the revised version of our study, we have undertaken the entire workflow using a new curated DAOx single mutant library. Additionally, we have made slight adjustments to the gate settings of the tyramide assay to ensure that no variants are excluded from our investigation (updated Fig 2C of the manuscript).
We acknowledge the reviewer's observation, and throughout the manuscript, we have experimentally demonstrated the significant dependence of the tyramide assay signal on the expression levels of the variants. As the reviewer rightly pointed out, we have also illustrated the similar correlation scores between activity and expression with the FOLDX predicted DDG and the disruptive effect that prolines typically exert when inserted in secondary structure regions of proteins.
To accurately identify hotspots and regions of the protein where mutations primarily influence activity, we have taken a comprehensive approach by cross-referencing our two datasets and

normalizing the activity to the expression (page 13, middle). This normalization provides a new statistic called 'normalized activity score', where changes in activity are less influenced by expression levels. Through this procedure, we have successfully dissected the activity-enhancing mutations from those affecting the underlying expression level, leveraging the DMS workflow described in the study. In the new figure S8C we present the heatmap of normalized activity scores. We made available also an interactive version of this heatmap at this link: heatmap_normalized_activity.

Is there some way the activity score could be normalized (divided by?) the expression score to provide such a quantity?

As mentioned above, we have included a new dataset in the paper, which involves normalizing the activity score to the expression score (page 13, middle). This normalization allows us to visualize the impact of pure activity-affecting mutations. In Figure S8B, we present a scatter plot depicting the relationship between normalized activity and expression score for all analysed variants. Additionally, in figure S8C we present the normalized activity score of all variants as a heatmap. To ensure accessibility and interactivity, we made the heatmap available online as an interactive tool (heatmap_normalized_activity).

I read through the section "Thermodynamic stability shapes both expression and activity landscapes," but didn't see any connection to thermodynamics. The section title may be more accurately described with "Biophysical properties" or "Physicochemical properties."

We appreciate the reviewer's accurate observation and valuable suggestions. In response, we have revised the title of the paragraph from "Thermodynamic stability shapes both expression and activity landscapes" to "Biophysical properties shape both expression and activity landscapes".

Line 347 (and below): the correlation coefficients may be missing a decimal? (Exp r=-26)

We have reviewed and updated the correlation coefficients in the main text.

- This is a very nice method that can plug into the many established coupled enzyme assays that utilize oxidases. The Asparginase in Fig S8 was great. In the Discussion, I think it would be worthwhile to list a few more diverse classes of enzymes that could (theoretically) be assayed with this method.

We have updated the conclusion following the suggestion of the reviewer as follows "Our workflow is compatible with studying enzymes that can be directly or indirectly (via enzymatic cascade) linked to the production of peroxide. This includes enzymes with immediate therapeutic relevance, such as Arginase, and Asparaginase (Fig. S8), as well as biocatalysts with diagnostic or industrial applications, like Glucose Oxidase."

Reviewer #2:

Summary

In this manuscript, Vanella, et al. develop a method called EP-seq to dissect the relationships between enzyme activity and stability in a high-throughput manner, building upon existing strategies for Yeast Surface Display (YSD), Deep Mutational Scanning (DMS), peroxidase-mediated radical labelling, and FACS. The authors create a DMS library of all possible single mutations of the enzyme D-amino Acid Oxidase (DAOx) and use two parallel assays: one to measure expression and another to estimate enzymatic activity. The resulting Expression Fitness and Activity Fitness scores are analyzed, compared, and mapped onto the three-dimensional structure of DAOx to explore their effects on its stability and activity.

I agree that there is a clear and pressing need for quantitative assays that can deconvolute enzyme activity and stability in high throughput. A fundamental understanding of this relationship would be transformative for biotechnology, medicine, and fundamental biology and would be generally interesting and impactful. My primary concern is that this assay cannot achieve the stated goal of this manuscript, which is to provide "biophysical insights into protein sequence, stability, and function" as there are caveats and biochemical oversimplifications in the creation and interpretation of this assay (see Major Points). In its current form and presentation herein, there is not enough evidence that this method alone has the quantitative power to deliver the biophysical insights that the authors seek. Perhaps the authors should consider presenting this method as a way for selection of more active and better expressed DAOx variants through multiple cycles of this assay, rather than as a tool for biochemical and biophysical dissection of activity-stability tradeoffs. The current motivation for the method requires a true deconvolution of activity and stability and data rooted in biochemical principals. For an engineering-focused motivation, detailed biochemical analyses could be performed on several final variants from the selection, whereas the current motivation of this method requires more controls to rigorously benchmark these assays against true biochemical and biophysical parameters. The comments below come from my perspective as an enzymologist and I sincerely hope that they are helpful to the authors.

We appreciate the comprehensive reviewer's feedback and acknowledge the concerns about the EP-seq workflow's ability to deconvolute activity and expression in bio-catalysts. Below, we've responded point by point to the comments trying to show that our method provides key insights into the enzyme's properties and supports further engineering for enhanced enzyme activity.

Major Points

1. What evidence do the authors have that the population of DAOx is correctly dimerized on the yeast surface? I am generally concerned about the use of a cytosolic, dimeric protein for these assays. For dimeric enzymes, proper folding is often coupled with dimerization and the folding of one subunit can be cooperative with the other […] More quantitatively, how much of the displayed DAOx is dimerized? The authors report that they measure a KM for their displayed WT DAOx with D-Ala of 5.01 ± 0.33 mM which is 6-fold higher than the reported KM of 0.8 mM. They do not show the curve this KM value was estimated from, making it challenging to assess the quality of this data. This KM discrepancy is concerning because it could be caused by incorrectly folded WT DAOx or the presence of a competitive inhibitor. Can the authors estimate the percentage of DAOx that is correctly dimerized based on the dimerization Kd and the surface concentration? Is there a way to measure this on the surface?

To support the idea that our wild-type DAOx maintains proper folding and dimerization on the yeast cell surface, we first conducted a comparative assessment of its catalytic features. We compared the enzyme displayed on yeast cells with the soluble counterpart expressed in BL21 *E. coli*. We purified the soluble enzyme using affinity chromatography followed by size exclusion chromatography (SEC) to obtain a soluble DAOx enzyme with high purity. These procedures followed prior reports (DOI: 10.1016/s0014-5793(02)03111-3) (figure 1). The soluble version of this enzyme is known to dimerize, and dimerization is required for catalytic activity.

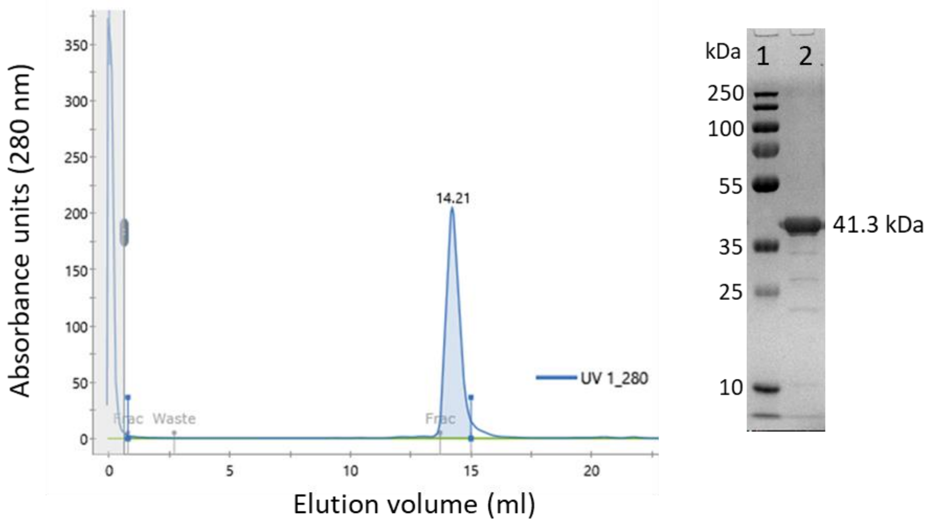

Figure 1: Purification of *Rg*DAOx Wild Type from BL21DE3 *E. coli*. (Left) Size exclusion chromatogram of ~0.5 mg *Rg*DAOx wild type enzyme. (right) SDS polyacrylamide gel electrophoresis analysis of ~2 µg of eluted protein.

We next selected six different substrates for DAOx, including D-alanine (D-ALA), D-methionine (D-MET), D-phenylalanine (D-PHE), D-glutamine (D-GLN), D-histidine (D-HIS) and D-threonine (D-THR). We assayed the activities of both yeast-displayed and soluble wild-type DAOx at various substrate concentrations (8 data points per substrate) to determine the Michaelis-Menten K_M constant for each substrate. K_M serves as an ideal kinetic parameter for comparison because it remains consistent regardless of the enzyme concentration tested in both systems. This is important because we cannot easily precisely quantify absolute concentration of enzymes displayed on the yeasts. The assays were performed at 25°C and pH 7.5 in a PBS buffer, utilizing the Amplex red assay at the same conditions as reported in the manuscript. Our results demonstrated a high degree of concordance between the K_M values calculated for the two enzymes expressed in different systems (soluble DAOx vs. yeast displayed DAOx). This comparison is presented in Table 1 below (for reviewer purposes). The table includes K_M values in millimoles per liter (mM) and the coefficient of determination (R^2), which indicates the goodness of fit of the Michaelis-Menten model for each experiment. In Figure 2 A and B (below, for reviewer purposes) we present all the Michaelis-Menten curves, showcasing the range of substrate concentrations used. If our yeast displayed enzymes were not correctly dimerized, we would have expected to observe differences in K_M. The strong correspondence in values between the K_M of the displayed enzyme and those of its soluble counterpart across all six tested substrates (Table 1) supports the hypothesis that wild-type DAOx adopts the correct conformation and maintains its native oligomerization state when expressed on the yeast cell surface.

In terms of comparison to literature, we attribute the differences in K_M for D-alanine for our yeast displayed DAOx in comparison to those reported for the soluble enzyme in the literature (https://doi.org/10.1074/jbc.M203946200) to two significant differences in experimental conditions. Firstly, we conducted our assay at pH 7.5, whereas the literature utilized pH 8.5. Secondly, the literature's DAOx activity assay took place under conditions of saturating oxygen, whereas we did not introduce additional oxygen into our reactions. We intentionally opted for these distinct conditions to better align with the pH requirements of the yeast cells used in our tyramide assay, as they needed to remain viable for subsequent regrowth after cell sorting. Additionally, this choice aimed to enhance the practicality of our assay. The manuscript was updated (Note 1, page S26) with the information regarding the newly calculated K_M value towards D-Alanine of the soluble and yeast displayed wild type DAOx.

Table 1. Michaelis-Menten constant K_M experimentally assayed for the yeast displayed and soluble form of the *Rg*DAOx wild type.

**Substrate**	**DAOx soluble K_M (mM)**	**R^2**	**DAOx displayed K_M (mM)**	**R^2**
D-ALA	**6.965 ± 0.4003**	0.9937	**7.316 ± 0.2816**	0.9973
D-MET	**2.214 ± 0.1608**	0.9850	**2.039 ± 0.1455**	0.9851
D-PHE	**2.497 ± 0.1033**	0.9950	**1.507 ± 0.05914**	0.9947
D-GLN	**16.12 ± 0.8415**	0.9961	**14.42 ± 0.8434**	0.9950
D-HIS	**3.612 ± 0.1662**	0.9942	**3.406 ± 0.2003**	0.9923
D-THR	**34.01 ± 2.745**	0.9883	**39.38 ± 1.548**	0.9975

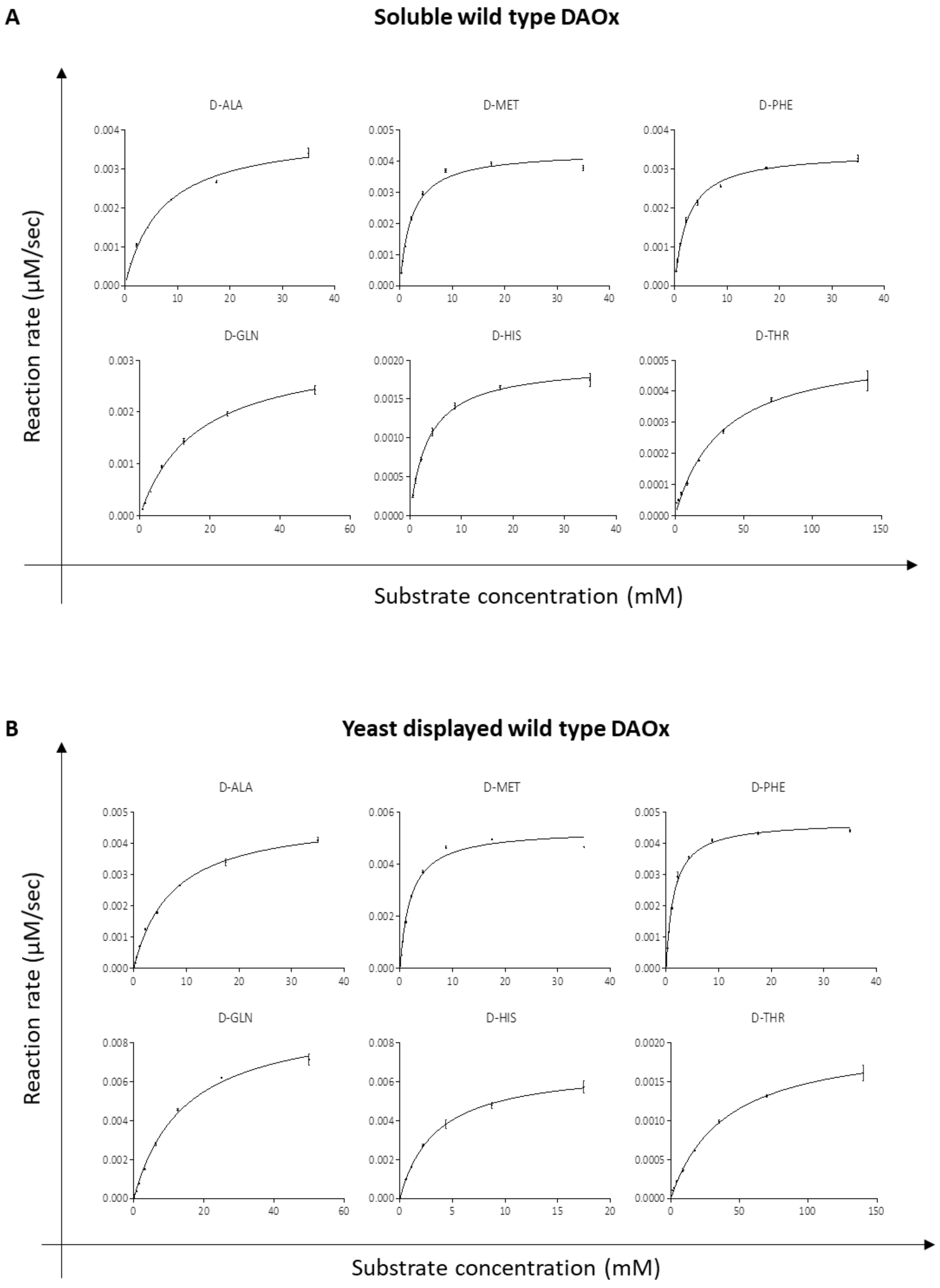

Figure 2. Initial velocities vs. substrate concentrations for yeast display and soluble DAOx. A Michaelis–Menten expression was used for fitting to determine K_M soluble wild type DAOx (A) and yeast displayed wild type DAOx (B) for 6 different D-amino acid substrates. Coefficients of determination (R^2) for each of the fitted datasets are reported in Table 1. K_M values for each substrate tested both with displayed and soluble DAOx are reported in Table 1.

Finally, as further support for correct dimerization on the yeast surface, we note that a comprehensive study on two *Rg*DAOx mutants (W243I and W243Y, https://doi.org/10.1111/j.1742-4658.2005.05083.x) provides details on how each of these mutations affects oligomerization. They reported that substituting tryptophan at position 243 with tyrosine increased the dimerization K_D, resulting in poor oligomerization at low enzyme concentrations, thereby reducing catalytic activity. Also, the mutation of tryptophan 243 to isoleucine disrupted oligomerization and was linked to loss of enzyme activity. Our sequencing-based assay aligns with these findings. We detected a significant deleterious effect for W243I (DMS expression score: -0.26, DMS activity score: -0.68). And with a less pronounced effect, a negative impact was also observed for W243Y (DMS expression score: 0.05, DMS activity score: -0.32).

Taken together, these data strongly support our wild type enzyme being properly folded and dimerized on the surface of the yeast.

And could the low activities of poorly expressed mutants be due to insufficient concentration to drive dimerization, rather than catalytic impairment?

It is possible that the expression of certain variants could be well below their dimerization K_D and this effect causes them to appear as both low activity and low expression variants. This represents a practical limitation of the assay. However, keep in mind that yeast surface display produces the enzymes in a confined space (i.e. 2D confinement on the cell wall) and this confinement effect would tend to drive dimerization rather than hinder it. If there are really insufficient amounts of particular enzyme variants on the surface to enable dimerization, we would observe this as both low expressing and low activity. By normalizing activity values to expression, as we have done in the updated version of the paper, these effects should be minimized. In any case they would only affect a small number of variants with low stability.

Since DAOx is trafficking through the yeast secretory pathway as a monomer (something that it hasn't evolved to do) and unfolded and misfolded proteins are degraded prior to display and dimerization on the surface, observed changes in abundance may not reflect true thermodynamic stability [i.e. the Gibbs free energy difference between the native dimerized state to the unfolded state(s)]. The expression measurement may instead represent the "stability" of an aberrant misfolded species or intermediate species not normally observed in high frequency when the protein folds in the cytosol. Since the trafficked protein is likely exposed to the protein degradation machinery as a monomer prior to surface display and dimerization, it is unlikely that the expression differences reported herein represent the thermodynamic stability of the native, dimerized state. Given these considerations, I think a rigorous definition of what the authors mean by stability is needed. Prior YSD studies referenced in this manuscript focused on TEM-1 beta-lactamase and Levoglucosan kinase, both monomeric enzymes (PMID: 28196882). The SARS-CoV-2 receptor binding domain (PMID: 32841599) is also monomeric.

We integrated our response to this part of point 1 along with our response to point 2. Please see below.

2. Furthermore, these two studies (PMID: 32841599 & 28196882) are cited in this manuscript to support the claim that YSD abundance of proteins is correlated to thermodynamic stability. This relationship between YSD abundance and thermodynamic stability, while likely holds generally, is also likely to break down in many instances and is not supported by enough in vitro data to quantitively understand the strength and shape of this relationship and where it does not hold. Situations where this relationship may not hold is with dimeric proteins with cooperative folding, as noted in the point above, and with proteins with complicated folding pathways where YSD abundance may be more sensitive to the kinetics of the process than the thermodynamics. What is known about the folding pathway of DAOx and is it likely to be affected by these other features? This comment could also be addressed by redefining the stability as “cellular stability” rather than thermodynamic stability, as the latter has a more rigorous, biochemical definition, or by measuring thermodynamic stability for a number of mutants to benchmark this assay.

In response to the reviewer's recommendation, we have refined our terminology and incorporated the following passage into the manuscript to clarify our interpretation of "folding stability" (page 4, top) “In this study, we use the term "folding stability" to describe the impact of mutations on the cellular stability of the target protein. This primarily relates to structural and thermodynamic stability, but can also include other factors like mRNA stability, efficiency of translation and secretion, and susceptibility to proteases degradation, all of which contribute to changes in the protein’s expression level.”

We acknowledge that validating the utility of yeast display as a platform for examining the effects of mutations on thermodynamic stability is best done on a case-by-case basis. But we also note that our manuscript offers multiple indications that substantiate the connection between the expression level of DAOx on the yeast surface and its folding stability.

For example, we employed the FOLDX program, a widely accepted in-silico tool for calculating protein folding energies and assessing the effects of point mutations on protein stability. Our in-silico predictions for mutations across the DAOx enzyme's entire sequence were compared to experimentally determined DMS expression and activity scores. This comparison revealed a strong correlation ($0.51 < \rho < 0.59$) between the in-silico predictions and our experimental results (Fig. S4C, D, manuscript). As pointed out by the same reviewer in point 4, this correlation between the FOLDX predicted values and both of our datasets supports the notion that folding stability plays a role in both enzyme expression and catalytic activity. In the revised version of the manuscript (page 13, middle), we have included data regarding the Spearman correlation coefficient (rho) between the FOLDX predicted values and the normalized activity. When we normalized activity to expression levels, the Spearman correlation coefficient decreased from 0.59 to 0.3. This demonstrates that FOLDX stability prediction does not correlate with normalized activity, but does correlate with expression levels. If we trust the accuracy of the *in silico* modelling, then this result supports the idea that expression fitness score, at least when analyzed on a large number of variants (i.e. population level analysis) is correlated with thermodynamic folding stability.

As additional evidence for the relationship between expression fitness and folding stability, we note for instance, how specific substitutions can influence expression fitness scores in our dataset. For example, prolines introduced into alpha helices were the most deleterious mutations found. Also, substitution of hydrophobic amino acids in the core of the protein resulted in low expression fitness.

Many of these effects are reported in quantitative terms in our paper. Collectively, these observations strengthen the argument that the expression level of DAOx in yeast can serve as overall reference to understand the impact of mutations on the overall folding stability of the protein. Nonetheless, in the interest of remaining cautious with our claims, we have modified the language and we no longer refer to 'thermodynamic stability' but rather use the term 'folding stability' and explain that this includes cellular stability factors along with other aspects (as stated above).

3. What sensitivity to differences in activity do the authors expect to get from an assay like this? What is the dynamic range? Over the 45-minute incubation with substrate, how much of the substrate do the authors expect to be consumed? Do they expect to still be in the initial rate regime for all variants? The authors have chosen an enzyme, DAOx, for this study that is exceptionally fast–kcat of 43,250 min-1 (PMID: 1348188). In Figure S1B, half a million cells displaying WT DAOx were used to benchmark the assay (although it is unclear at what concentration) and the reaction already appears to be out of the initial linear range after 10 minutes. The challenge of a pooled assay like this is that faster or better expressed enzymes will consume substrate quickly, starving lower-expressed or less active variants of substrate and artificially lowering their observed activity. This is perhaps why the correlation between the single clone score and the Activity Fitness score (Fig 2G) is unremarkable and is worse than that observed for the Expression Fitness assay. In addition, 35 mM D-Ala is only 7-fold above the observed KM. One could imagine that mutations would increase KM from 5 mM to something where 35 mM is no longer saturating, adding an additional level of complexity to the interpretation of this data.

To address these concerns and enhance the robustness of our method, while revising the manuscript we further optimized the tyramide assay and repeated our sorting/sequencing experiments. The first observation of the reviewer is that based on the K_M for D-Ala for the yeast displayed DAOx (K_M = 6.965 ± 0.4003 mM), the substrate concentration used in the prior version of the manuscript (35 mM D-Ala) may have been too low to ensure the enzyme was working at V_{max} throughout the entire tyramide assay incubation period. To address this, we have increased the D-Ala substrate concentration to 130 mM for all the sorting/sequencing experiments in the updated version. This adjustment maintains D-Ala at nearly 20 times the calculated K_M. Additionally, in this updated version we introduced a viscosity enhancer in the form of 0.75% wt/vol sodium alginate into the reaction mixture. This polymer additive was found to improve the sensitivity and specificity of our assay by slowing down molecular diffusion. Furthermore, we increased the concentration of fluorescent tyramide by a factor of 10 and we reduced the cell concentration to 500 cells/µL. These modifications collectively contributed to higher sensitivity and specificity of the tyramide assay compared to the previous conditions. With these modifications, we found that the overall signal intensity was higher and the unspecific background signals were reduced. We additionally conducted a more thorough exploration of the reaction kinetics by observing the reaction at multiple time points (Figure 3, for review only). After careful analysis, we determined that a 20-minute incubation time was sufficient. These adjustments and findings have been updated and added to the revised version of the manuscript.

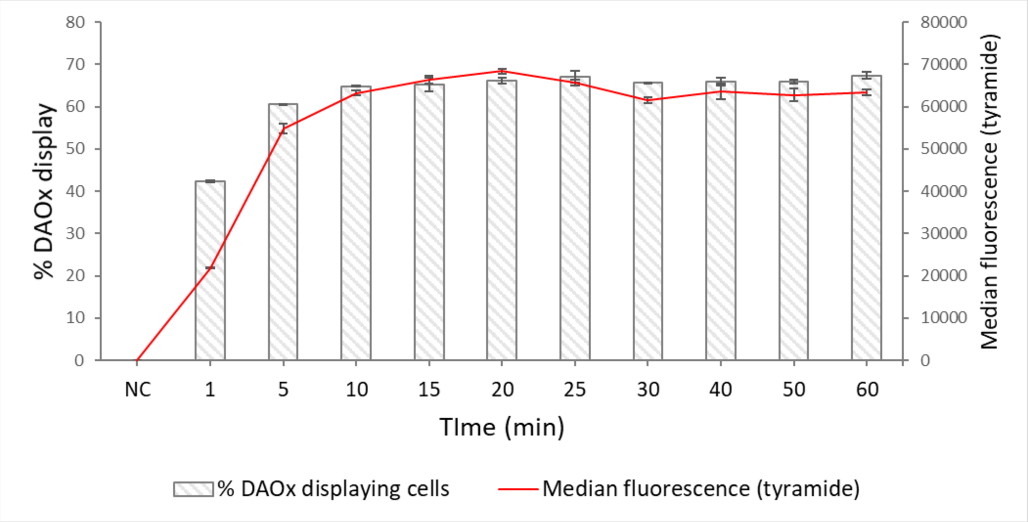

Figure 3. Time course of tyramide reaction with the updated conditions. Here are reported both the percentage (%) of cell population acquiring the tyramide signal over time (bars) and the median fluorescence of the tyramide positive population (red line). The reaction appears to be completed between 15 to 20 min of incubation, therefore 20 min was selected as incubation time in the updated version of this assay.

To address the reviewer's comment on the dynamic range of the single-cell tyramide labelling assay, we present results here which take advantage of the promiscuity of DAOx. DAOx is active on a wide range of D-amino acid substrates, and for each substrate the enzyme exhibits a distinct set of Michaelis-Menten parameters (K_M and k_{cat}). To explore the dynamic range of our tyramide assay, we selected six substrates: D-alanine (D-ALA), D-methionine (D-MET), D-phenylalanine (D-PHE), D-glutamine (D-GLN), D-histidine (D-HIS), and D-threonine (D-THR). According to the literature and based on our own previous results, these substrates were expected to show relatively low K_M values, making them easily saturable in aqueous medium, as well as a broad range of turnover numbers (k_{cat}). We experimentally determined turnover numbers for each of these six substrates using soluble wild type DAOx purified from BL21 *E. coli* (refer to Figure 1). The turnover numbers for each of the substrates we tested are presented in Table 2, and were found to cover a range of k_{cat} values from 1.9 to 74 s^{-1}. This range of turnover numbers served as a reference for standardizing the dynamic range of our single-cell tyramide labelling assay.

Table 2: Turnover number (k_{cat}) experimentally determined for 6 of RgDAOx substrate using soluble DAOx wild type enzyme expressed in BL21 bacterial cells. For each substrate tested are also presented the K_M values for the DAOx displayed enzyme and the concentration of initial substrate used in the tyramide reaction in order to guarantee saturating condition of the enzyme along the 20 minutes incubations of the reaction.

**Substrate**	**k_{cat} (s^{-1}) of soluble DAOx**	**K_M of displayed DAOx (mM)**	**[Substrate] (mM) during tyramide labeling reaction**
No substrate (Neg.control)	0	-	-
D-THR	1.9	39.4	375.2
D-GLN	11.2	16.1	134

D-HIS	33.8	3.6	187.6
D-PHE	57.2	1.6	93.8
D-ALA	68.0	7.3	93.8
D-MET	74.3	2.0	93.8

Given the strong agreement between the kinetic values obtained for the soluble and displayed enzymes, as shown in Figure 2, we conducted our tyramide assay using saturating concentrations of each substrate for the displayed enzyme (Figure 4,A). We anticipated achieving similar turnover numbers to those experimentally measured for the soluble enzyme.
This approach allowed us to detect the median fluorescence and determine the percentage of the population exhibiting green fluorescence following tyramide labeling. We tested for correlation between the tyramide signal generated from yeast labelling and the measured k_{cat} values on the soluble enzymes, thereby showcasing the assay's sensitivity to differences in k_{cat} and offering a range of turnover numbers where our assay could be effectively applied.

The result of this approach demonstrates strong linear correlation between k_{cat} measured on the various substrates and the fluorescence signal on the yeast cells expressing DAOx wild type after the tyramide assay (R^2=0.93) (Figure 4, for review purposes). This robust relationship highlights the direct correspondence between the enzyme's speed and the tyramide fluorescence signal that is generated under saturating substrate conditions. Moreover, this correlation defines a linear range of k_{cat} values, spanning from 1.9 to 74 s^{-1}, over which our assay can discriminate activity. Using the tyramide labelling technique, we were able to detect differences in k_{cat} as low as 1.9 s^{-1}, which was determined as the limit of detection (LOD) of the tyramide assay using the formula LOD = (3.3* average standard deviation – intercept)/slope.

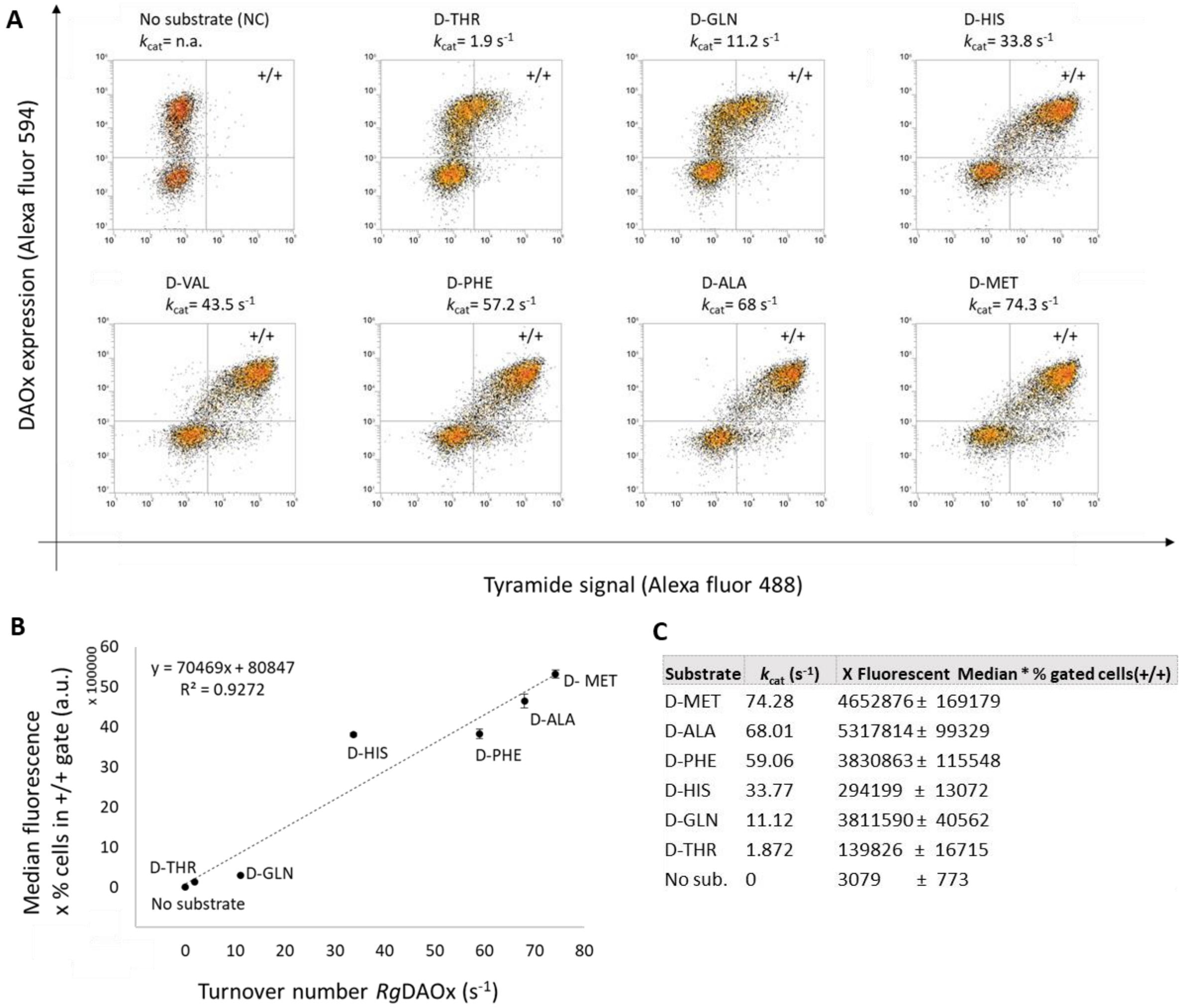

Substrate	k_{cat} (s^{-1})	X Fluorescent Median * % gated cells(+/+)
D-MET	74.28	4652876 ± 169179
D-ALA	68.01	5317814 ± 99329
D-PHE	59.06	3830863 ± 115548
D-HIS	33.77	294199 ± 13072
D-GLN	11.12	3811590 ± 40562
D-THR	1.872	139826 ± 16715
No sub.	0	3079 ± 773

Figure 4. Detailed characterization of the sensitivity of the tyramide assay in detecting differences in enzyme activity (k_{cat}) under saturating substrate conditions. (A) Flow cytometry density plots depict a population of yeast cells expressing wild-type DAOx that underwent the tyramide labelling assay with various substrates under saturating concentrations. Cells acquiring the green tyramide signal and shifting toward the gate +/+ were further analysed. (B) A linear correlation is shown between the tyramide labelling signal and turnover number (k_{cat}) of soluble DAOx purified from *E. coli.* For the tyramide labelling signal, we used the median fluorescence of the positive tyramide population multiplied by the percentage of cells in the gate +/+. (C) Details on the k_{cat} values and median fluorescence values multiplied by the percentage of cells in the gate +/+, and shown in panel B.

To complete the response to the point 3 we note here that the correlation between the single clone activity tested and the DMS activity fitness score reported in Figure 2G (manuscript) improved from r=0.65 (p-value=0.024) to r=0.96 (p-value=1.3e-06) as reported in the updated version of Figure 2 of the main manuscript. We attribute the improvement of this correlation coefficient to the higher quality of the DMS score detected by repeating the entire workflow with a curated library of DAOx variants and using the optimized tyramide assay protocol discussed above.

4. Related to comment #3, the authors do not normalize their activity fitness assay by abundance. This means they cannot distinguish between variants that are well expressed, but comparatively less active from those that are more active but comparatively less expressed. This convolution is likely why the authors see nearly as good of a correlation between FoldX ΔΔG predictions and their Expression Fitness score as they see for FoldX ΔΔG predictions and their Activity Fitness scores. The authors do not show if there is a correlation between their Expression Fitness score and their Activity Fitness scores, but this would be expected purely from the lack of abundance normalization for their activity measurements. It is critical for the stated goal of this manuscript for the assay to be able to deconvolute activity and expression. Consider normalizing activity scores by expression score to distinguish between activity and stability in analysis.

We have included a new dataset in the manuscript, which involves normalizing the activity score to the expression score as requested by the reviewer. As pointed out by the reviewer this normalization allows us to visualize the impact of mutations on activity without the confounding effect of enzyme abundance. In Figure S8A of the updated supplementary information for publication, we now present a scatter plot depicting the relationship between activity and expression score for all analysed variants. Additionally, we have incorporated a visualization of the normalized activity vs. expression score (Fig. S8B manuscript) and as a heatmap (Fig. S8C manuscript). To ensure accessibility and interactivity, we have made the heatmap available online as an interactive tool (heatmap_normalized_activity).

5. Related to comments #3 and #4, the most novel aspect of this manuscript is the activity assay. The gold-standard for enzyme activity measurement is cellular expression, purification, and measurement of kcat and KM for enzyme variants. This assay should be benchmarked against this gold standard.

In response to point 3, we have conducted a comprehensive characterization of our assay, comparing its performance to the k_{cat} values of purified soluble DAOx in presence of different substrates. This analysis revealed a strong correlation between the enzyme velocity and the tyramide signal acquired by the cells and detected through flow cytometry (see above). We believe this addresses at least some of the concerns raised in point 5 of the reviewer's feedback, where further assay benchmarking against catalysis values was requested. We are actively utilizing the insights gained from the presented assay to explore various substrates for DAOx and different enzymes expressed on yeast cell surfaces. We anticipate that forthcoming work will provide further evidence of the assay's reliability in accurately detecting reaction velocity.

6. The authors state: “Only 5 positions (~1%) were found with positive activity fitness and negative expression fitness (Fig. 5A, top left).” This result could be due to the convolution of expression and activity in their Activity Fitness assay and/or due to the pooled format of this activity assay where fast, well-expressed variants compete with lowly-expressed variants for substrate.

We fully concur with the reviewer's observation. Indeed, we have identified only a limited number of variants where activity could be detected at low expression levels. As the reviewer rightly points out, this dependence of activity on expression levels is a crucial factor. Our enzyme proximity sequencing workflow is specifically designed to address this aspect. The workflow encompasses two distinct and

parallel measurements on the impact of mutations on both expression and activity. By conducting these parallel experiments, we can subsequently compare the results and differentiate activity scores from expression scores. In the updated version of the manuscript, we have performed the normalization of activity to expression level, which addresses this comment.

7. The authors state: "This shows that mutations at the catalytic site tended to harm activity but improve stability (avg. Exp=-0.068, +12%, avg. Exp all=-0.077, Fig. 3DI), suggesting the enzyme pays a thermodynamic price to remain catalytically active under conditions of functional selection." The authors are describing a well-documented phenomenon stemming from the seminal work of Shoichet and Matthews (PMID: 7831309) and is one of the foundational studies that suggested activity-stability tradeoffs. This should not be presented as a new model and Shoichet and Matthews should be cited.

We modified text according to the reviewer's suggestions and included the missing reference. This section now reads as follows (page 13, top): "This revealed how mutations at the catalytic site tended to harm activity but improve stability (avg. Exp=-0.068, +12%, avg. Exp all=-0.077, Fig. 3DI), supporting a well-documented phenomenon on the thermodynamic price paid by an enzyme to remain catalytically active under conditions of functional selection[4]."

8. I do not see the noteworthiness of the following statement since it would be predicted from the conservation: "Noteworthy was an N-terminal region (residues 8-32) found to be highly intolerant to amino acid substitutions. This suggests it plays a role in folding stability and could act as an N-terminal intra-molecular chaperone48. The Rossmann fold is highly conserved in this region".

The text was modified in accordance with the reviewer's suggestion as follow (page 9, bottom): "The expression heatmap (Fig. 2I) reveals patterns of higher and lower tolerance for mutation along the DAOx sequence, discussed in detail below. The N-terminal region (residues 8-32) was found to be highly intolerant to amino acid substitutions. This suggests it plays a role in folding stability and could act as an N-terminal intra-molecular chaperone."

9. It is unclear whether weighting scores by the number of cells is statistically appropriate. I am concerned that in an assay like this, with variations in number of cells and surface expression between different DAOx variants, a weighted mean would skew the interpretation of the data (the authors note this as a positive though, saying "This allowed us to place more emphasis on fitness values that were represented by a higher number of cells in an experimental replicate, resulting in final consensus fitness scores that were calculated with higher confidence"). Can the authors explain in more detail in the methods why this is a sounder statistical metric for this assay rather than a simple mean and doesn't serve to minimize assay error?

We appreciate the reviewer's observation, which prompted us to reassess our approach critically.

First, in the revised version of the manuscript, we have removed the weighted scores and we now employ and show a more conventional linear correlation coefficient between the standard fitness values of the two replicates for both the expression and activity assays. The updated values, as presented in the new Figure 2B and D (manuscript), indeed demonstrate a high linear correlation

coefficient for both replicates in both assays (Expression r=0.94, Activity r=0.96). The high correlation we see with the replicates (now unweighted) is due to the improved parameters of the tyramide assay, as well as to the improved quality of the mutant library achieved through content curation and size reduction to approximately 200,000 variants. This size reduction made the library more manageable and has increased reproducibility in sorting and analysis.

However, to calculate the final consensus score for each variant we still used a weighted mean, where the number of cells acted as the weighting factor. This choice is motivated by the fact that variants represented by a greater number of cells provide more reliable fitness scores. This is to say, we have more confidence in fitness scores that were generated by larger numbers of observations. It's important to emphasize that, given the substantial correlation between biological replicates, this approach doesn't substantially change the result as compared to using a simple mean value of fitness scores from experimental replicates (we have compared them). Nevertheless, for the best precision in single variant scoring, particularly for variants that exhibit high variability among replicates, we prefer to use the weighted average when calculating the final consensus score.

10. I am concerned that the conclusions of this paper are too general or already known. For example, the authors state "This indicates that in order for a variant to be catalytically active, it must first be stably expressed and secreted to the cell wall. We therefore observed an expression level-dependency of the catalytic activity." This is a requirement of their assay and central to all of enzyme kinetics (i.e., more enzyme, greater velocity), not a new result or conclusion. The authors state: "We found that WT residues in close proximity to the FAD cofactor in the active site tended to destabilize the enzyme, and could be mutated to enhance folding stability, however this stability was achieved at the cost of catalytic activity." This general concept was described nearly 20 years ago by Shoichet and Matthews (see comment #7). In the context of a new system like DAOx, it would be generally interesting if the authors were able to provide mechanistic and biophysical explanations for why there is an activity-stability tradeoff at these positions.

In response to the reviewer's comments, we have adjusted the manuscript's conclusions. Our emphasis is now on the workflow, which facilitates high-throughput enzyme analysis. We acknowledge that the notion of the stability-function trade-off in enzymes has been also previously studied and demonstrated in some natural catalysts, as noted by the reviewer. Nonetheless, the scale of our dataset and our ability to observe these effects on pooled data for a large number of enzyme variants is new and noteworthy. To provide more mechanistic and biophysical explanations for why there is an activity stability tradeoff at the core catalytic residues interacting with FAD cofactor, we examined expression and normalized activity in relation to hydrophobicity changes resulting from mutations at these positions (Supporting Figure S8D, E). We calculated delta hydrophobicity as the difference between the hydrophobicity of the wild-type amino acid and the substituent. Negative delta hydrophobicity indicates an increase in hydrophobicity, while positive values indicate decreased hydrophobicity. These results show how increase in hydrophobicity at the core of the protein significantly correlate with increase of expression while having on average neutral to negative effects on the catalytic activity. The amino acids substituted at the analysed sites can increase stability through hydrophobic effects, at the cost of catalytic activity.

Minor Points

• Use of word "Fitness": we are unsure if it is appropriate to use the word fitness in this context (i.e., activity fitness score) as the central premise of this paper is to decouple activity from a fitness selection

We employ the term "fitness" to mean phenotypic fitness in an evolutionary sense. Variants with high fitness are more likely to be propagated into future generations, in our case due to the influence of an artificial selection pressure imposed by a laboratory screening condition (display level or catalytic activity sorted on FACS). In our case, we used "expression fitness" to indicate the level of expression and "activity fitness" to refer to the level of activity exhibited by the enzyme variants.

• Fig 1, 2, 3: consider switching away from red-green colorscale for accessibility

We appreciate the reviewer's comments. In the revised version of the manuscript, we have included all heatmaps in an interactive version and in a colorscale that is accessible to individuals with color blindness. These can now be accessed at the following links: heatmaps_expression_activity, heatmap_normalized_activity.

• Fig 2 F/H: what do the vertical dashed lines represent?

The dashed lines represent the range of fitness score for synonymous variants respectively for the expression fitness (Fig. 2F) and activity fitness (Fig. 2H). The missing information was inserted in the caption of Figure 2.

• Fig 2 I/K: heatmaps as very difficult to interpret in current formatting, consider allocating more space to them and rotating them horizontally (perhaps J/L could be moved to supplement?)

To enhance accessibility and improve the readability of the heatmaps, we have incorporated interactive heatmaps in the revised version of the manuscript. These can now be accessed at the following links: heatmaps_expression_activity, heatmap_normalized_activity.

• Page 7, para 2: who's whose

This typographical error was corrected.

• Fig 4B: Consider coloring D-ala backbone differently from FAD backbone

We have modified the color of the D-Alanine backbone as suggested by the reviewer.

• Page 12, para 1: "This demonstrates structure-function tradeoffs at play" should this say stability-function tradeoff?

We have changed the text following the reviewer suggestion.

• Page 13, para 1: how is distance to interface calculated? Can this be 0?

The residues found at the interface with direct contact with the other monomer have a value of distance to interface equal to 0. The distance to interface was calculated as below and as described in the methods section of the manuscript.

"Residues involved in the monomer-monomer contact at the dimer interface of DAOx dimeric structure (PDB:1C0P) were selected through the pyMol tool "InterfaceResidues" (https://pymolwiki.org/index.php/InterfaceResidues). Coordinates of the alpha carbons (Cα) of interface residues were extracted from the file 1C0P.pdb and the distance between each residue of the structure to the closest interface Cα was calculated."

• Page 13, para 1: "quaternary" is unnecessary

We have changed the text following the reviewer suggestion.

REVIEWERS' COMMENTS

Reviewer #1 (Remarks to the Author):

The authors have addressed all my comments.

Reviewer #2 (Remarks to the Author):

Vanella, et al have thoroughly responded to my comments and I believe the revised manuscript has been greatly improved. My remaining comments are mostly for clarity.

1. The authors replaced "thermodynamic stability" with "folding stability", but they also use the term "structural stability". What is the difference between structural and folding stability? If they are intended to be the same term, please update for consistency.
2. The authors use the section title "Stability activity trade-offs along natural evolution of DAOx." I do not see how these experiments relate to features of natural DAOx evolution besides being one mutational step away from their WT sequence.
3. On page 7, the authors write: "This result supports the idea that a baseline level of structural stability is required for an enzyme to be catalytically active, and that part of the EP-Seq activity score is attributable to effects of mutations on the structural stability (and therefore expression) of DAOx". Since the coupling of folding and activity is a basic tenet of biochemistry, I would revise it as follows: "As a baseline level of structural stability is required for an enzyme to be catalytically active, this result supports that part of the EP-Seq activity score is attributable to effects of mutations on the structural stability (and therefore expression) of DAOx."
4. On page 14, the authors say "All these observations comprehensively show how functional constraints have shaped the evolution of DAOx over time". The authors do not show this directly or comprehensively as they only make single mutations on one DAOx ortholog. This statement should be softened.
5. In response to my major point #1, the authors note "It is possible that the expression of certain variants could be well below their dimerization KD and this effect causes them to appear as both low activity and low expression variants. This represents a practical limitation of the assay." As this paper presents a new method, this practical limitation should be noted in the main text of the manuscript for completeness. Generally a sentence or two that outlines the benefits AND limitations of this assay is necessary for readers.

General comment for all the reviewers:

We express our gratitude for the valuable inputs and time invested by the reviewers. Their constructive feedbacks have greatly contributed to enhancing the overall quality of our work. In the following sections we respond point by point to the comments of the reviewers. The reviewer's queries are highlighted in black while our corresponding responses are in blue.

Reviewer #1 (Remarks to the Author):
The authors have addressed all my comments.

We appreciate the reviewer's positive assessment of our work, and we're happy that our responses effectively addressed all the questions raised.

Reviewer #2 (Remarks to the Author):
Vanella, et al have thoroughly responded to my comments and I believe the revised manuscript has been greatly improved.

We are pleased that our responses have effectively addressed the reviewer's inquiries, leading to an improvement in the overall clarity and quality of the work.

My remaining comments are mostly for clarity.

1. The authors replaced "thermodynamic stability" with "folding stability", but they also use the term "structural stability". What is the difference between structural and folding stability? If they are intended to be the same term, please update for consistency.

The text and Figure 1 were updated by replacing "structural stability" with "folding stability" as suggested by the reviewer.

2. The authors use the section title "Stability activity trade-offs along natural evolution of DAOx." I do not see how these experiments relate to features of natural DAOx evolution besides being one mutational step away from their WT sequence.

According to the reviewer's comment we have rephrased the title of the section to "Identification of Stability activity trade-offs regions in DAOx"

3. On page 7, the authors write: "This result supports the idea that a baseline level of structural stability is required for an enzyme to be catalytically active, and that part of the EP-Seq activity score is attributable to effects of mutations on the structural stability (and therefore expression) of DAOx". Since the coupling of folding and activity is a basic tenet of biochemistry, I would revise it as follows: "As a baseline level of structural stability is required for an enzyme to be catalytically active, this result supports that part of the EP-Seq activity score is attributable to effects of mutations on the structural stability (and therefore expression) of DAOx."

We have modified the text following the reviewer's suggestion

4. On page 14, the authors say "All these observations comprehensively show how functional constraints have shaped the evolution of DAOx over time". The authors do not show this directly or

comprehensively as they only make single mutations on one DAOx ortholog. This statement should be softened.

As suggested by the reviewer the text was softened as follow “These observations suggest that functional constraints have contributed to shape the evolution of DAOx over time, and provide direct and clear evidence of an activity-stability tradeoff.”

5. In response to my major point #1, the authors note “It is possible that the expression of certain variants could be well below their dimerization KD and this effect causes them to appear as both low activity and low expression variants. This represents a practical limitation of the assay.” As this paper presents a new method, this practical limitation should be noted in the main text of the manuscript for completeness. Generally a sentence or two that outlines the benefits AND limitations of this assay is necessary for readers.

In response to the reviewer's suggestion, we have made slight modifications to the text to incorporate information on how the method has specific constraints. These include the functional expression of the enzyme on the cell surface and the direct or indirect linkage of enzymatic activity to peroxide production.

The updated text reads as follows: 'Our workflow is compatible with enzymes that can be functionally displayed on the cell surface and whose activity can be directly or indirectly (via enzymatic cascade) linked to the production of peroxide.

We opted for the term "functional expression," which automatically includes various limitations related to enzyme expression level. This encompasses ensuring a minimal concentration for proper oligomerization in cases where enzyme function depends on oligomerization and guaranteeing a minimal enzyme expression level to lead to sufficient activity for the detection of tyramide fluorescence linked to the cell surface.
